# Expression Elements Derived From Plant Sequences Provide Effective Gene Expression Regulation and New Opportunities for Plant Biotechnology Traits

**DOI:** 10.3389/fpls.2021.712179

**Published:** 2021-10-22

**Authors:** Jennifer P. C. To, Ian W. Davis, Matthew S. Marengo, Aabid Shariff, Catherine Baublite, Keith Decker, Rafaelo M. Galvão, Zhihuan Gao, Olivia Haragutchi, Jee W. Jung, Hong Li, Brent O'Brien, Anagha Sant, Tedd D. Elich

**Affiliations:** ^1^Bayer Crop Science, Chesterfield, MO, United States; ^2^GrassRoots Biotechnology, Durham, NC, United States; ^3^Monsanto Company, Research Triangle Park, Durham, NC, United States; ^4^Pairwise Plants, Durham, NC, United States; ^5^Duke University, Office for Translation and Commercialization, Durham, NC, United States; ^6^LifeEDIT Therapeutics, Durham, NC, United States

**Keywords:** gene expression, plant biotechnology, promoter, intron, 3′ UTR, transcription, expression elements, optimized

## Abstract

Plant biotechnology traits provide a means to increase crop yields, manage weeds and pests, and sustainably contribute to addressing the needs of a growing population. One of the key challenges in developing new traits for plant biotechnology is the availability of expression elements for efficacious and predictable transgene regulation. Recent advances in genomics, transcriptomics, and computational tools have enabled the generation of new expression elements in a variety of model organisms. In this study, new expression element sequences were computationally generated for use in crops, starting from native Arabidopsis and maize sequences. These elements include promoters, 5′ untranslated regions (5′ UTRs), introns, and 3′ UTRs. The expression elements were demonstrated to drive effective transgene expression in stably transformed soybean plants across multiple tissues types and developmental stages. The expressed transcripts were characterized to demonstrate the molecular function of these expression elements. The data show that the promoters precisely initiate transcripts, the introns are effectively spliced, and the 3′ UTRs enable predictable processing of transcript 3′ ends. Overall, our results indicate that these new expression elements can recapitulate key functional properties of natural sequences and provide opportunities for optimizing the expression of genes in future plant biotechnology traits.

## Introduction

Innovations in plant biotechnology have delivered ways to enhance agricultural productivity and sustainability, as well as improve crop quality to meet the farmer and consumer needs (Datta, 2013; Aldemita et al., 2015). With growing demands for productivity and quality for a growing world population, as well as growing pressures from insect pests, weeds, and climate change on crop production (Tilman et al., 2011; FAO, 2017), new traits with increasing variety are critical to meet these increasing needs (Huang et al., 2015; Ricroch and Hénard-Damave, 2016; Li et al., 2020). Newer plant biotechnology products require trait combinations, also known as trait stacks, to provide multiple trait solutions within one crop (Que et al., 2010; Huang et al., 2015). Sequence redundancy among stacked traits has been identified as a potential risk factor in transgene expression instability (Vaucheret et al., 1998; Kooter et al., 1999; Fagard and Vaucheret, 2000). Hence, diversifying sequences to avoid redundancy, including in expression elements and transgene coding sequences, is important for reducing this risk and maintaining stability and efficacy in plant biotechnology traits. In addition, new biotechnology product concepts may require new modes of expression control to achieve trait efficacy. Altogether, these combined trends lead to an increasing need for diversified and optimized expression solutions.

The availability of efficacious and diverse gene expression elements has been identified as a key bottleneck for developing new biotechnology traits in plants (Que et al., 2010; Nuccio, 2018). For both protein-coding and noncoding transgenes, expression is conferred by a combination of key gene expression elements that are collectively called a gene expression cassette. The gene expression cassette requires the following key components: a promoter, a 5′ untranslated region (UTR), a 3′ UTR, and optionally, one or more introns. The promoter and 5′ UTR enable the assembly of the transcription initiation machinery and recruit RNA polymerase II to the transcription start site (TSS) (Smale and Kadonaga, 2003; Hetzel et al., 2016), thus directing the transcription of the intended coding or noncoding trait gene-of-interest. The 5′ UTR also recruits ribosomes to initiate translation of the coding sequence from transcribed mRNAs (Sonenberg and Hinnebusch, 2009). The 3′ UTR defines the cleavage and polyadenylation of the pre-mRNA, while also contributing to transcriptional initiation and elongation of coding or noncoding sequences (Proudfoot, 2004). The gene expression cassette can also include one or more introns that are transcribed as part of the pre-mRNA and are spliced out during pre-mRNA processing (Lorkovic et al., 2000). Introns can contribute to expression regulation (Le Hir et al., 2003), including increasing expression through a mechanism known as an intron-mediated enhancement (Rose and Beliakoff, 2000; Rose, 2018).

Novel expression elements have been generated for transgenes in plants using a variety of methods, including leveraging cisgenic or transgenic sequences from plants or other species, mutation of such sequences, combinatorial arrangement of fragments or motifs from these sequences, and *de novo* design methods (Venter, 2007; Peremarti et al., 2010; Nuccio, 2018). Outside of directly sourcing native sequences from plants or other species, most novel expression elements described in the literature have been generated from native sequences by using mutational or combinatorial methods (Liu et al., 2014; Rushton, 2016; Grant et al., 2017; Maruyama et al., 2017; Belcher et al., 2020). Recent advances in genomics, combined with machine learning and other computational tools, have offered new opportunities to learn from native genomic sequence datasets for applications in modulating gene expression (Camacho et al., 2018; Gilman et al., 2019; Hollerer et al., 2020). Recent publications have reported *de novo* design of promoters in various model systems, including bacteria and yeast (Kotopka and Smolke, 2020; Wang et al., 2020), and have primarily focused on short sequences, comprising the core promoter. The development of short core promoter sequences in plants, with demonstrated expression in transient reporter systems, has also been reported (Jores et al., 2021).

In this study, we have characterized a set of new expression elements that are computationally derived from native plant sequences. Our promoters include the core promoter and extend upstream to include sequences that confer unique expression profiles, and downstream to include the 5′ UTR. In addition to promoters, we have developed introns and 3′ UTRs and demonstrated *in planta* function for all three classes of elements. We present data from expression characterization of these elements in stably transformed plants, including a detailed analysis of the transcripts produced. Our data demonstrate that these expression elements effectively leverage motifs learned from native sequences to drive reporter gene expression across a variety of plant tissues. With these characteristics, our computationally derived expression elements show promise for delivering increased predictability and tunability for optimizing plant biotechnology traits.

## Materials and Methods

### Plant Sequence Training Set Identification

**Figure 1:**
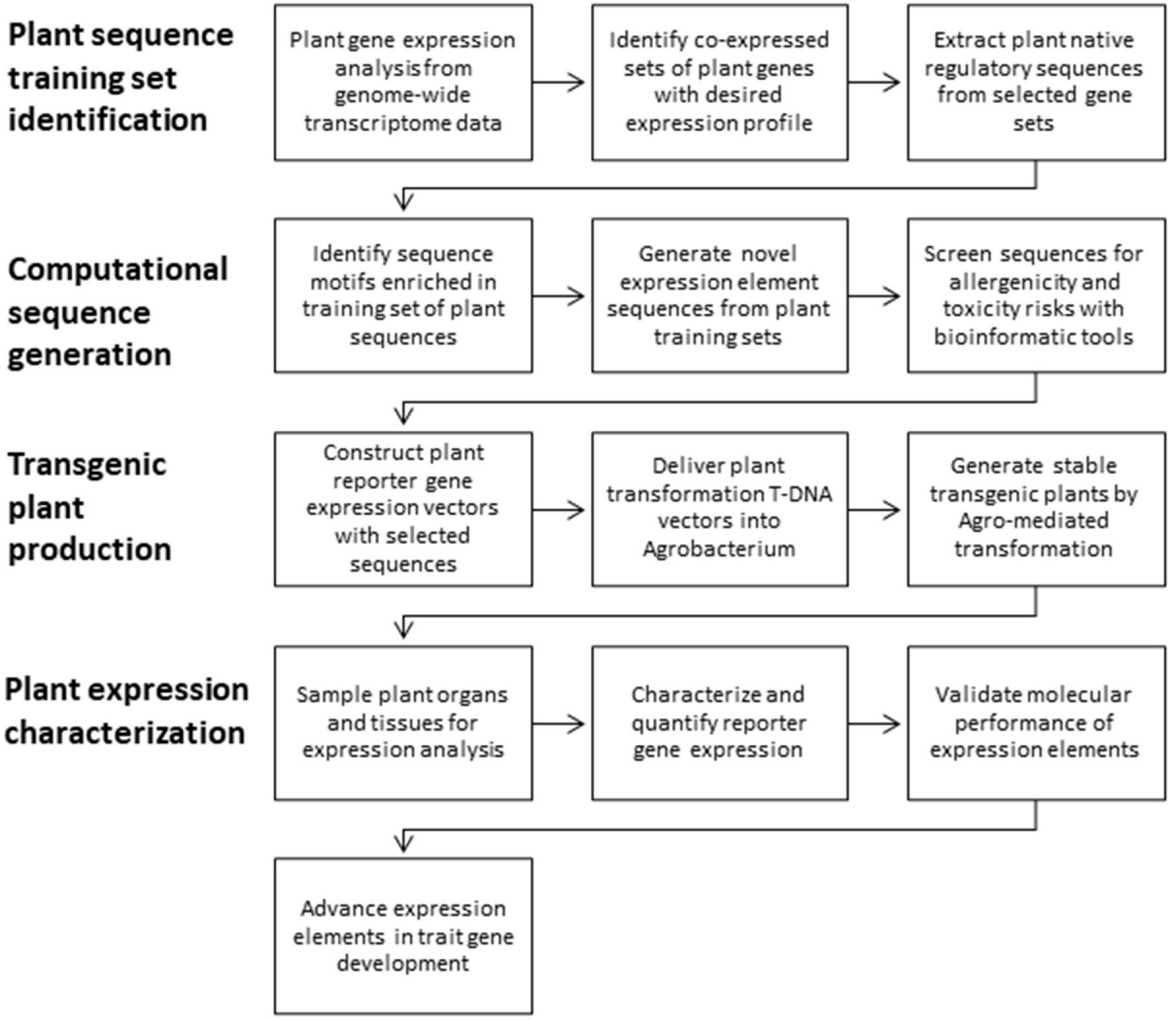
Overview of expression element development and testing.

Expression elements were computationally derived from nucleotide sequences from native genes with desired expression properties (Figure 1). First, sets of co-expressed Arabidopsis or maize genes were identified from genome-wide transcriptome data across a variety of tissue types and developmental stages in *Arabidopsis thaliana* (Schmid et al., 2005; Brady et al., 2007) and *Zea mays* (Schnable et al., 2009). Gene expression values for Arabidopsis microarray and maize RNAseq were first calculated for each gene and each tissue by using established methods, gcRMA (Wu et al., 2004) and FPKM (Mortazavi et al., 2008), respectively. Co-expressed gene sets were then identified by unsupervised clustering of calculated gene expression values (Brady et al., 2007). The expression elements characterized in this study were derived from sequences of 102 *Arabidopsis* constitutively expressed genes (At.GSP442, At.GSI17, and At.GSI21), 129 Arabidopsis leaf-preferred genes (At.GSP571 and At.GSP576), and 1,461 maize-expressed genes (Zm.GST7). Sequence training sets from co-expressed native genes were extracted from Arabidopsis TAIR9 (Swarbreck et al., 2008) or Maize B73 RefGen_v1 (Schnable et al., 2009) genome assemblies with adjusted annotations based on EST mapping (Alexandrov et al., 2008; Schnable et al., 2009; Soderlund et al., 2009; Troukhan et al., 2009). Plant native regulatory sequences were extracted from these Arabidopsis and maize gene annotations to collect training sets as follows: promoters and 5′ UTRs (1,000 bp upstream of the transcription start site (TSS) to 50 bp downstream of TSS), introns (sequence between 5′ and 3′ splice sites with flanking 5 bp), and 3′ UTRs (−400 to +200 bp relative to the polyadenylation site).

### Computational Sequence Design

Position-specific enrichment of motifs in the sequence training sets was identified by POWRS as previously described (Davis et al., 2012). The identified putative motifs are predicted to contribute to the expression pattern and/or molecular function. The sequence training sets and putative motifs were used to train a proprietary machine learning algorithm to generate new expression element sequences. The promoters and 5′ UTRs were named GrassRootsPromoters (GSPs), the introns were named GrassRootsIntrons (GSIs), and the 3′ UTRs were named GrassRootsTerminators (GSTs). The GSPs, GSIs, and GSTs described in this study were approximately 500 bp, 300 bp, and 300 bp, respectively. These sizes are in general agreement with literature estimates of the sizes of these elements (Korkuc et al., 2014; Jafar et al., 2019). Additional design constraints were applied to reduce the risk of unintended molecular function. GSPs include a short leader sequence at the 3′ end to promote transcript processing and translation initiation of the resulting mRNA. To avoid unintended coding sequences, start codons (ATG) were avoided downstream of the predicted TSS. GSIs also include flanking exonic sequences for efficient splicing and avoiding consensus splice site sequences (Lorkovic et al., 2000) between the intended splice sites to reduce the risk of alternative splicing. As an additional precaution, bioinformatic analysis was performed to meet regulatory requirements for safety assessment of plant biotechnology products (Codex_Alimentarius, 2009), and only expression elements that met these requirements were advanced for the optimization of plant biotechnology traits.

All expression element nucleotide sequences characterized in this study are provided in Supplementary Material S1.

#### Expression Element Motif Variants

Known key motifs for molecular function of native expression elements were modified to transversions in GSPs, GSIs, and GSTs. These motifs included TATA boxes for GSPs (Zhu et al., 1995), 5′ and 3′ splice sites (Lorkovic et al., 2000), as well as intron-mediated enhancement (IME) motifs for GSIs (Rose, 2008), and the near-upstream element (NUE) and T-rich tracts for GSTs (Li and Hunt, 1995). The sequences of expression element variants with motif mutations characterized in this study are provided in Supplementary Material S1.

### Transgenic Plant Generation

GSPs, GSIs, GSTs, and their motif variants were tested in the context of a transgenic β-Glucuronidase (*GUS*) reporter gene from *Escherichia coli*. The functional gene expression unit, as a combination of gene expression elements and reporter gene-coding sequence, is described as a gene expression cassette. A reference gene expression cassette (cassette 1 in Table 1) was generated to comprise a series of plant native expression elements operatively linked together with the GUS-coding sequence, including (from 5′ to 3′) promoter and leader sequence from *Arabidopsis thaliana CYTOCHROMEC OXIDASE* gene (AT4G37830) (*At.Cyco*_promoter_leader), the first intron of the same gene (*At.Cyco*_intron) inserted within the 5′ UTR, coding sequence from *Escherichia coli* GUS gene with an inserted intron from *Solanum tuberosum* light-inducible gene (Ec.uidA+St.LS1), and the 3′ UTR from *Gossypium barbadense* Fiber Late gene (*Gb.Fbl2*). DNA fragments of the GSPs, GSIs, and GSTs were generated by synthesis and sequence verified (Bio Basic, Markham, ON, Canada). To generate expression cassettes to test the novel sequences, the corresponding functional element(s) from the reference cassette were replaced with GSPs, GSIs, or GSTs fragments. The expression cassettes were inserted into a binary plant transformation vector and verified by sequencing. The T-DNA vectors were transformed into *Agrobacterium* and introduced into *Glycine max* (A3555 germplasm) by *Agrobacterium*-mediated transformation. Transformed plants were assayed for GUS insertion copy number by DNA TaqMan assays. Transformed plants that had a single copy of the GUS transgene and normal morphological characteristics were selected for further tissue sampling and analysis.

**Table 1:** A list of expression cassettes.

**Cassette**	**Promoter**	**Intron**	**GOI**	**3^**′**^ UTR**
1	At.Cyco_promoter_leader	At.Cyco_intron	Ec.uidA+St.LS1	Gb.Fbl2
2	At.GSP442	At.Cyco_intron	Ec.uidA+St.LS1	Gb.Fbl2
3	At.GSP571	At.Cyco_intron	Ec.uidA+St.LS1	Gb.Fbl2
4	At.GSP576	At.Cyco_intron	Ec.uidA+St.LS1	Gb.Fbl2
5	At.GSP442_TATA	At.Cyco_intron	Ec.uidA+St.LS1	Gb.Fbl2
6	At.GSP571_TATA	At.Cyco_intron	Ec.uidA+St.LS1	Gb.Fbl2
7	At.GSP576	At.GSI17	Ec.uidA+St.LS1	Gb.Fbl2
8	At.GSP571	At.GSI21	Ec.uidA+St.LS1	Gb.Fbl2
9	At.GSP576	At.GSI17_IME	Ec.uidA+St.LS1	Gb.Fbl2
10	At.GSP576	At.GSI17_splicesite	Ec.uidA+St.LS1	Gb.Fbl2
11	At.GSP571	-	Ec.uidA+St.LS1	Gb.Fbl2
12	At.GSP571	At.GSI21_IME	Ec.uidA+St.LS1	Gb.Fbl2
13	At.GSP571	At.GSI21_splicesite	Ec.uidA+St.LS1	Gb.Fbl2
14	At.GSP571	At.Cyco_intron	Ec.uidA+St.LS1	Zm.GST7
15	At.GSP571	At.Cyco_intron	Ec.uidA+St.LS1	Zm.GST7_NUE
16	At.GSP571	At.Cyco_intron	Ec.uidA+St.LS1	Zm.GST7_T-rich_tracts

Details of the expression cassettes characterized in this study are listed in Table 1. Sequences of the reference expression cassette and component elements are provided in Supplementary Material S1.

### Plant Expression Characterization

The following organs were sampled from plants in the T0 generation at vegetative (V) and reproductive (R) developmental stages: V3 stage leaf and root; V5 stage leaf (source and sink) and root; R1 stage leaf (source and sink), root, and flowers; R3 stage pod and immature seed; and R5 stage seed cotyledon. Plant developmental stages were identified as previously described (Licht, 2014).

#### GUS Reporter Analysis

To assay quantitative GUS enzymatic activity, approximately 50 mg of fresh weight tissue was lyophilized and powdered. Total protein was extracted from the powdered tissue using a 500- to 800-μl 100-mM KPO4 (pH 7.4) extraction buffer (supplemented with 1-mM NaEDTA, 0.1% lauryl sarcosine, 0.1% Triton 100 X, 0.05% glycerol, 2% PVP, 10-mM β-mercaptoethanol, and 0.2-mM PMSF). Total protein concentration was determined using the Bradford protein assay per instructions of the manufacturer (BIO-RAD Life Science, Hercules, CA, USA). To assay for GUS activity, 1-3-μg total protein extract was incubated with 50-nmol 4-methylumbelliferyl-β-D-glucopyranosiduronic acid (MUG) substrate (Sigma Aldrich, St Louis, MO, USA) in a 50-μl reaction at 37°C for 1 h. The reaction was stopped by the addition of 350-μL 0.2-M sodium carbonate. The fluorescence product was measured with excitation at 365 nm, emission at 445 nm using a FLUOstar Omega microplate reader (BMG Labtech, Cary, NC, USA), then converted to pmol 4 MU with a standard curve and normalized to total protein. GUS enzyme activity was reported as pmol 4 MU/μg total protein/h. Statistical analysis was performed by *t*-tests between sample groups to determine *p*-values. A Bonferroni-type procedure (Benjamini and Hochberg, 1995) was used to determine *p*-value cutoffs for multiple comparisons and control for a false discovery rate <0.05. Comparisons that met the significance criteria were reported in the results with the adjusted *p*-value thresholds.

To conduct qualitative expression analysis of the transformed plants, fully expanded leaves, roots, and flowers were collected from plants at the R1 stage. Leaf cross sections were cut to 90–120-micron thickness using a sliding microtome (Leica Biosystems, Buffalo Grove, IL, USA). Roots were cut manually to collect 0.5–1-mm thick sections in the mature zone. Flowers were bisected to enable staining buffer access. Leaf sections, root sections, and bisected flowers, were submerged in GUS staining solution: 1-mg/ml X-Gluc (5-bromo-4-chloro-3-indolyl-b-glucuronide) (Sigma Aldrich, St Louis, MO, USA), 25-μM potassium ferricyanide, 2.5-μM potassium ferrocyanide, 0.05% Triton X-100 (v/v) in a 50-mM potassium phosphate buffer (pH 7.4). The tissues were incubated in the staining solution at 37°C for 5 h. Destaining was performed by incubating in 70% EtOH: glacial acetic acid (1:1 v/v) overnight, followed by 70% EtOH wash. The tissues were imaged under a stereodissecting microscope (Nikon Instruments, Melville, NY, USA) for flowers, or a compound microscope (Nikon Instruments, Melville, NY, USA) for leaf and root cross sections to detect cell-type-specific expression patterns.

#### Transcript Characterization

##### Transcription Start Site Mapping by 5′ RACE

RNA was extracted from flash frozen soybean V3 or R1 leaf tissue *via* RNeasy Plant Mini kit (QIAGEN, Germantown, MD, USA), followed by RNase-free DNaseI (Ambion) treatment and cleanup by RNA Clean and Concentrator-5 kit (Zymo Research, Irvine, CA, USA) per instructions of the manufacturers. 5′ Rapid Amplification of cDNA Ends (RACE) was performed by using the First Choice RLM-RACE kit (Ambion-Thermo Fisher Scientific, Waltham, MA, USA). 5′ ends of the target cDNA were amplified by nested PCR with two pairs of the adaptor and gene-specific primers. Gene-specific primers were designed for the GUS reporter-coding sequence. PCR products were TA-cloned *via* Topo TA Cloning kit (pCRIII) (Invitrogen-Thermo Fisher Scientific, Waltham, MA, USA) and sequenced using an M13 reverse sequencing primer. Transcription start sites were identified based on the alignment of reads, containing the 5′ adapter to the expression cassette sequence.

##### Intron Splicing and 3′ Polyadenylation Characterization by Sequencing

Ribonucleic acid was extracted and purified as described for 5′ RACE above. Amplicons were generated by using the SMARTer® RACE 5′/3′ Kit (Takara Bio USA, Mountain View, CA, USA). In brief, cDNA was generated from total RNA by using modified oligo(dT) primers. Amplicons were created using 25 cycles of touch-down PCR with a gene-specific primer and Universal Primer A Mix. The PCR product was purified by using SeqPurebeads (Biochain, Newark, CA, USA) and verified by using a fragment analyzer (Agilent Technologies, Santa Clara, CA, USA). Final libraries were created using the Nextera DNA Flex Library Prep Kit (Illumina, San Diego, CA, USA) for tagmentation and sequencing primer addition. The final amplification and adapter addition were performed with Kappa HiFi HotStart ReadyMix (Roche Sequencing and Life Science, Wilmington, MA, USA). Libraries were pooled and sequenced with a NextSeq 500/550 mid-output kit v2.5, 300 cycles (Illumina, San Diego, CA, USA). The reads were adaptor trimmed (Trim Galore) (Krueger, 2012) and mapped to the expression cassette sequence by using a splice-aware aligner (STAR) (Dobin et al., 2013). For each expression cassette, transcripts from at least three independent transgenic events were characterized, and all uniquely mapped reads were pooled across events for the analysis. For intron-splicing analysis, exon-exon junctions were identified, and the number of reads spanning exon-exon junctions and exon-intron junctions was quantified and compared to evaluate frequencies of expected splicing, unexpected splicing, and unspliced transcripts. 3′ polyadenylation sites were identified based on the alignment of reads containing the 3′ sequencing adapter. Primer sequences used for amplicon generation are provided in Supplementary Material S2.

##### Reverse Transcriptase Quantitative Polymerase Chain Reaction (RT-qPCR) Characterization of GUS Transcript and Read-Through

Ribonucleic acid was extracted from flash frozen soy V3 or R1 leaf tissue with Tri-reagent (Sigma Aldrich, St Louis, MO, USA) and purified by Zymo Direct-zol 96 RNA kits (Zymo Research, Irvine, CA, USA) with Turbo DNase treatment (Thermo Fisher Scientific, Waltham, MA, USA). RT-qPCR assays were performed using ABI Fast 1-Step Mix (Thermo Fisher Scientific, Waltham, MA, USA) on Applied Biosystems 7900 HT instrument per instructions of the manufacturer. TaqMan primer-probe sets were designed to the GUS-coding sequence, normalizing genes, and a read through amplicon downstream of the 3′ UTR (Supplementary Material S2). Relative expression of the GUS and read-through transcripts were calculated and normalized by using the 2[-ΔΔC(T)] method (Schmittgen and Livak, 2008). The % read through was calculated as a percentage of read-through transcripts as compared to GUS. Statistical analysis was performed by *t*-tests with adjustments to control for false discovery in multiple testing as described above for GUS quantitative analysis. Primer and probe sequences are provided in Supplementary Material S2.

## Results

### New Expression Elements Were Generated From Native Sequence Training Sets

New expression elements were generated and advanced for trait gene optimization by the following framework in four main parts (Figure 1). First, training sets of native plant sequences are collected. These training sets are nucleotide sequences from plant genes that demonstrate the desired expression profile (e.g., constitutive or leaf preferred) and the intended expression regulatory function (e.g., promoters, introns, or 3′ UTRs). Second, using these nucleotide sequence training sets, new expression element sequences are generated using computational tools to recapitulate the properties represented in the training set. Third, the novel sequences are introduced into plants in the context of transgenic expression cassettes to test for function in stably transformed plants. Fourth, these new expression elements are characterized in detail *in planta* to evaluate the expression profiles and molecular function. Finally, the expression elements that meet the criteria for effective expression and molecular function are then advanced to enable trait gene optimization and development.

New expression elements characterized in this study include GrassRootsPromoters and 5′ UTRs (At.GSP442, At.GSP571, and At.GSP576) and GrassRootsIntrons (At.GSI17 and At.GSI21) that are derived from Arabidopsis training sets, as well as a GrassRootsTerminator (Zm.GST7) that is derived from a maize training set.

To show that the novel sequences are diversified from the training sets, both new and plant expression elements in the test cassettes were searched against the Arabidopsis and maize genomes by BLAST (Supplementary Material S3). As expected, both the native Arabidopsis sequences *At.Cyco*_promoter_leader and *At.Cyco*_intron aligned with their respective genomic source sequences with nearly complete and identical matches to the TAIR9 reference genome (E-score 0 and 1e-176). In contrast, BLAST searches with the novel sequences, as well as the native cotton sequence Gb.Fbl2_3′ UTR, only generated matches to the Arabidopsis genomic sequence with low alignment scores (≤46.4) and low significance (*E*-score > 1e-5). The sequence alignments only gave fragmented matches with short stretches of continuous sequence identity ≤22 bp. Similarly, the GSPs, GSIs, and GSTs, as well as all of the native Arabidopsis and cotton expression elements, also only generated matches to the maize genomic sequence with low alignment (≤51.8) and significance scores (*E*-score >1e-4). These results indicate that the novel sequences bear no significant sequence identity to Arabidopsis or maize genomic sequences that were used in their development. The BLAST analysis further demonstrated that the native plant expression elements analyzed here also bear no significant sequence identity to the genomic sequences of other plant species analyzed here.

### GSPs Deliver Effective Gene Expression Profiles and Predictable Transcript Initiation

**Figure 2:**
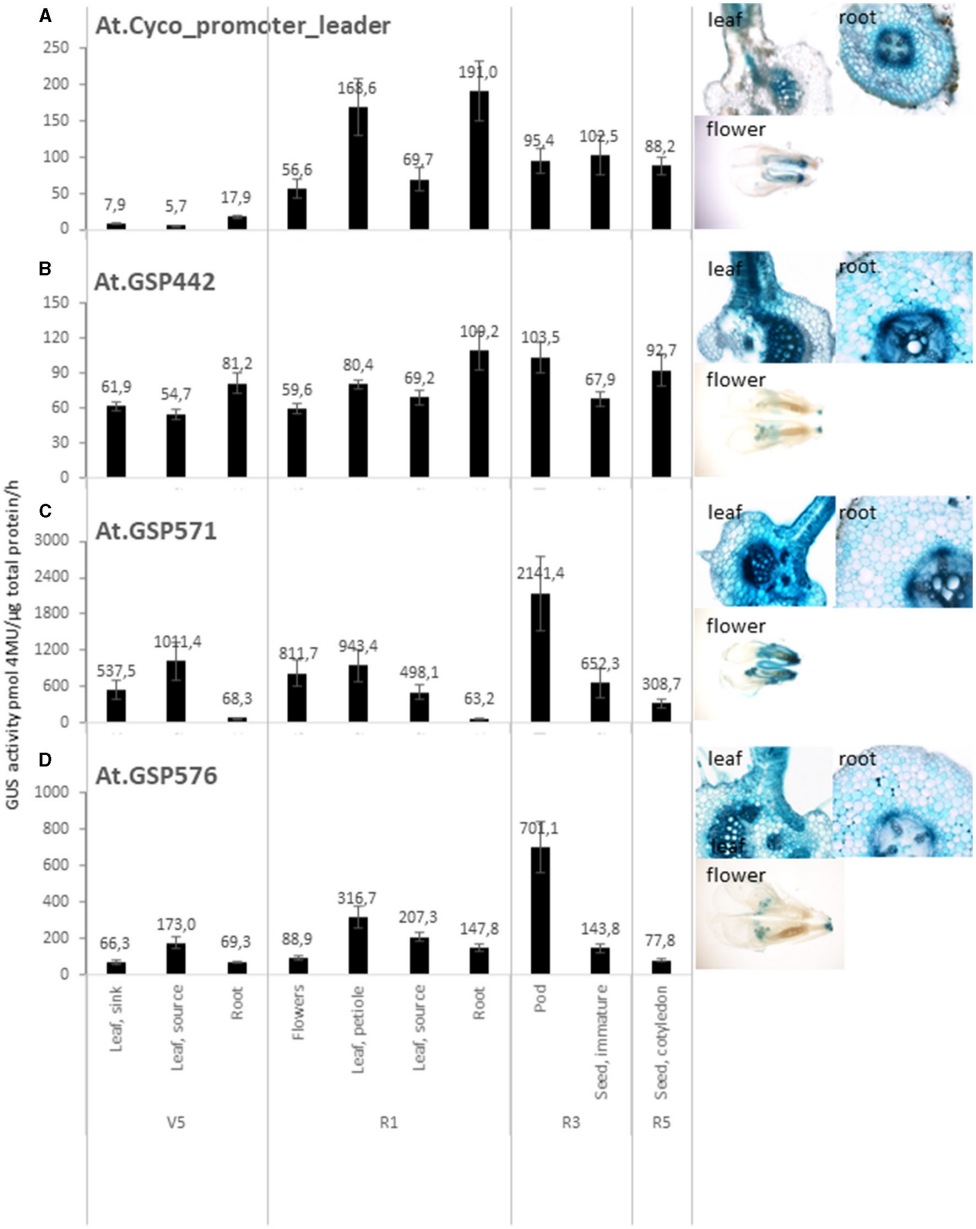
GSPs drive effective gene expression. Quantitative and qualitative analysis of GUS reporter genes driven by promoter and leaders from At.Cyco **(A)**, At.GSP442 **(B)**, At.GSP571 **(C)**, and At.GSP576 **(D)**. Left panels: Quantitative analysis of GUS reporter gene activity was performed on transgenic soybean plants across multiple tissues, including leaf, root, flowers, pods, and seeds over vegetative (V5) and reproductive (R1, R3, and R5) stages. GUS enzyme activity on MUG substrate was normalized to total protein and reported as pmol 4-MU/μg total protein/h. At least six independent transgenic events were analyzed, and the data are reported as the mean with standard error. Right images: Qualitative analysis was performed on leaf, root, and flowers at the R1 stage. Tissue cross**-**sections (leaf and root) or whole mount (flower) were incubated with X-Gluc substrate to produce blue staining where GUS enzyme activity was detected. At least five independent transformation events were imaged, and one representative image is shown.

To evaluate the function of the new promoters *in planta*, stably transformed transgenic soybean plants with β-Glucuronidase (*GUS*) reporter gene from *Escherichia coli* were generated. All promoter and 5′ UTR leader sequences were tested in the context of the same expression cassette, where each promoter and 5′ UTR leader sequence were operatively linked to the *At.Cyco*_intron, Ec.uidA+St.LS1, and the Gb.Fbl2_3′ UTR (Table 1). The *At.Cyco* promoter_leader (Figure 2A) and CaMV.35S_promoter_leader (Supplementary Material S7) were used as references for comparison with the GSPs.

To evaluate the overall performance of these computationally derived promoters, a larger set of GSPs was generated from the training set of Arabidopsis leaf-preferred genes, including At.GSP571 and At.GSP576, and was tested with the GUS reporter in stable soy transformants. Overall, 43% of the 156 GSPs tested demonstrated medium to super-high levels of average leaf tissue expression as intended. The highest expression levels detected were comparable to *CaMV.35S*, while low-leaf expression was detected in the rest of the GSPs, indicating that this computational approach can generate a useful range of transgene expression that can be utilized for different plant biotechnology traits. We focused the detailed characterization efforts on a subset of expression elements.

The expression profile of expression cassettes with *At.Cyco* promoter_leader, At.GSP442, At.GSP571, and At.GSP576 (Table 1, cassettes 1, 2, 3, and 4) was characterized in detail across vegetative and reproductive stages in leaf, root, flower, pod, and seed. All four promoter cassettes showed measurable GUS reporter activity across multiple tissue types and developmental stages (Figures 2A–E), with the exception that the *At.Cyco*_promoter cassette expression at V5 stage leaf was below the limit of quantification (<20-pmol 4-MU/μg total protein/h). GUS-staining images at the R1 developmental stage in cross-sections of source leaf and root, as well as whole mount flowers, corroborated the quantitative GUS activity detected. Moreover, this staining showed broad GUS expression across multiple cell types in the leaf and root, although *At. Cyco* promoter expression in the leaf was concentrated in vascular tissues. Overall, the range of GUS activity detected across tissue types from the GSP cassettes was comparable or higher than *At. Cyco*, indicating that these computationally derived promoters can effectively drive gene expression.

While all four promoters tested showed detectable activity, the expression profile of each promoter was unique. The *At. Cyco* promoter showed a developmentally regulated profile (Figure 2A). GUS expression was significantly higher in both source leaf and root in R1 than in V5 (*p* <0.05), where the GUS activity increased from the limit of quantification at V5 to 69.7 ± 15.6-pmol/μg total protein/h and 191. ± 41.4-pmol/μg total protein/h in R1 source leaf and root, respectively. At.GSP442 demonstrated low to medium expression with a broad profile that was root enhanced (Figure 2B). GUS activity in roots was measured at 81.2 ± 8.8-pmol/μg total protein/h and 109.2 ± 16.6-pmol/μg total protein/h at V5 and R1 stages, which was significantly higher than in source leaf at both stages (*p* < 0.05) by 48 and 58%, respectively. At.GSP571 and At.GSP576 both showed high-expression levels that were leaf and pod enhanced (Figures 2C,D). For both At.GSP571 and At.GSP576, the highest GUS activity was found in the pod wall, measured at 2,141.4 ± 616.3-pmol/μg total protein/h and 701.1 ± 141.9-pmol/μg total protein/h, respectively. The detected GUS expression in pod walls was significantly higher than immature seed dissected out of the pod at the same R3 stage and the R5 stage (*p* < 0.05), with a greater than 3-fold difference in GUS activity. At.GSP571 showed generally above ground-preferred expression, with significantly higher expression in various leaf and flower tissues than in root tissues at both V5 and R1 stages (*p* < 0.05). At.GSP576 showed a similar above ground-preferred expression similar to At.GSP571, albeit with overall lower levels of expression across above ground tissues compared with At.GSP571. Significant differences were also observed in V5 leaf, R1 flowers, and R3 and R5 seed tissues (*p* < 0.025), ranging from a 2- to 9-fold difference. In particular, expression in flowers for At.GSP576 is significantly lower than leaf tissues sampled at the same developmental stage (*p* < 0.05), indicating a difference in tissue-specific expression profiles between At.GSP571 and At.GSP576. Overall, our data demonstrate that these new promoters can direct diverse expression levels and tissue specificity.

**Figure 3:**
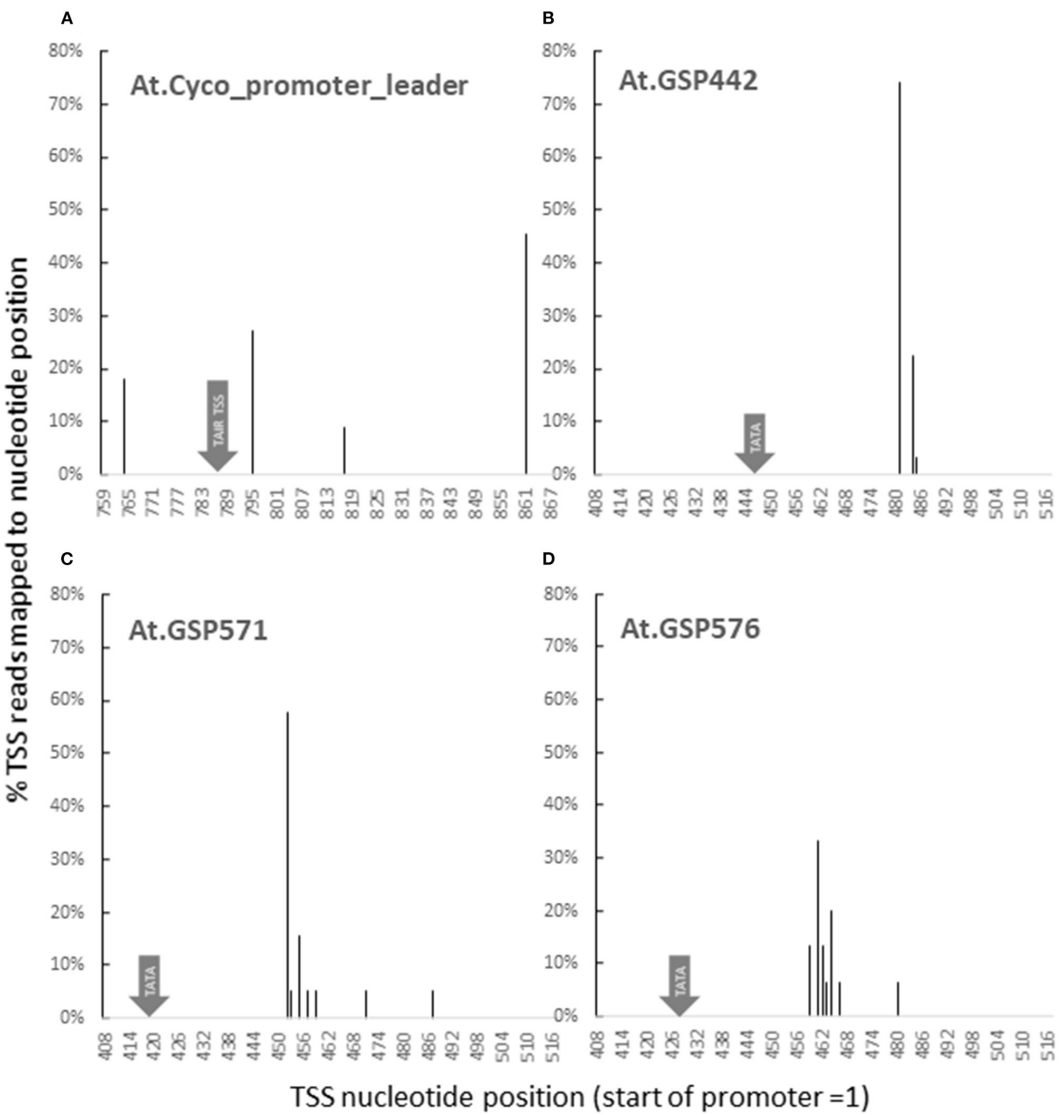
GSPs initiate transcription as predicted. Transcription start sites (TSS) for GUS reporter genes driven by promoters and leaders from At.Cyco **(A)**, At.GSP442 **(B)**, At.GSP571 **(C)**, and At.GSP576 **(D)** were identified by 5′ RACE. 5′ ends of GUS cDNA fragments generated from leaf RNA were captured and sequenced. At least two independent transgenic events were characterized to produce > 20 reads. Sequencing reads were mapped to promoters to identify the TSS. The resulting sequencing reads were pooled to report the TSS distribution as % reads mapped to each nucleotide position. Nucleotide positions are reported with the 5′ most position of the promoter = 1.

To assess whether the key molecular function of promoters followed predictions, 5′ RACE was used to map transcription start sites (TSS) for *At.Cyco*_promoter and the three GSPs. For *At. Cyco*, transcription initiation was detected at four sites across a 100-bp region (Figure 3A). Interestingly, none of the four detected sites coincided with the TSS annotated in TAIR9 or TAIR10 at 787 (position numbered with 5′ end of promoter = 1). The closest TSS detected was at 795, which was similar to the plant Initiator element (Inr) (Nakamura et al., 2002; Hetzel et al., 2016). The dominant transcription start site detected was the most downstream of the four at 861. All four TSS's for *At.Cyco*_promoter_intron were located ~30-bp downstream of an AT-rich region, which is consistent with previous reports of transcription initiation in relation to TATA-like sequences (Zuo and Li, 2011; Hetzel et al., 2016). For all three GSPs, 90% transcription initiation was detected within a 10-bp window, with dominant transcription start sites detected at positions 481, 452, and 462, for At.GSP442, At.GSP571, and At.GSP576, respectively (Figures 3B–D). The sequences at all three GSP TSSs were similar to the Arabidopsis Inr-like consensus sequence (Hetzel et al., 2016). These transcription initiation sites were also predictably located ~30 bp downstream of the intended TATA box, similar to endogenous plant promoters. These dominant and narrow TSS peaks were also found to be consistent with results from high throughput sequencing of 5′ transcript ends (Supplementary Material S4). Overall, these TSS mapping results for At.GSP442, At.GSP571, and At.GSP576 are consistent with the function of this intentionally placed TATA box in directing transcription initiation through interaction with RNAPol II and other cellular machinery.

**Figure 4:**
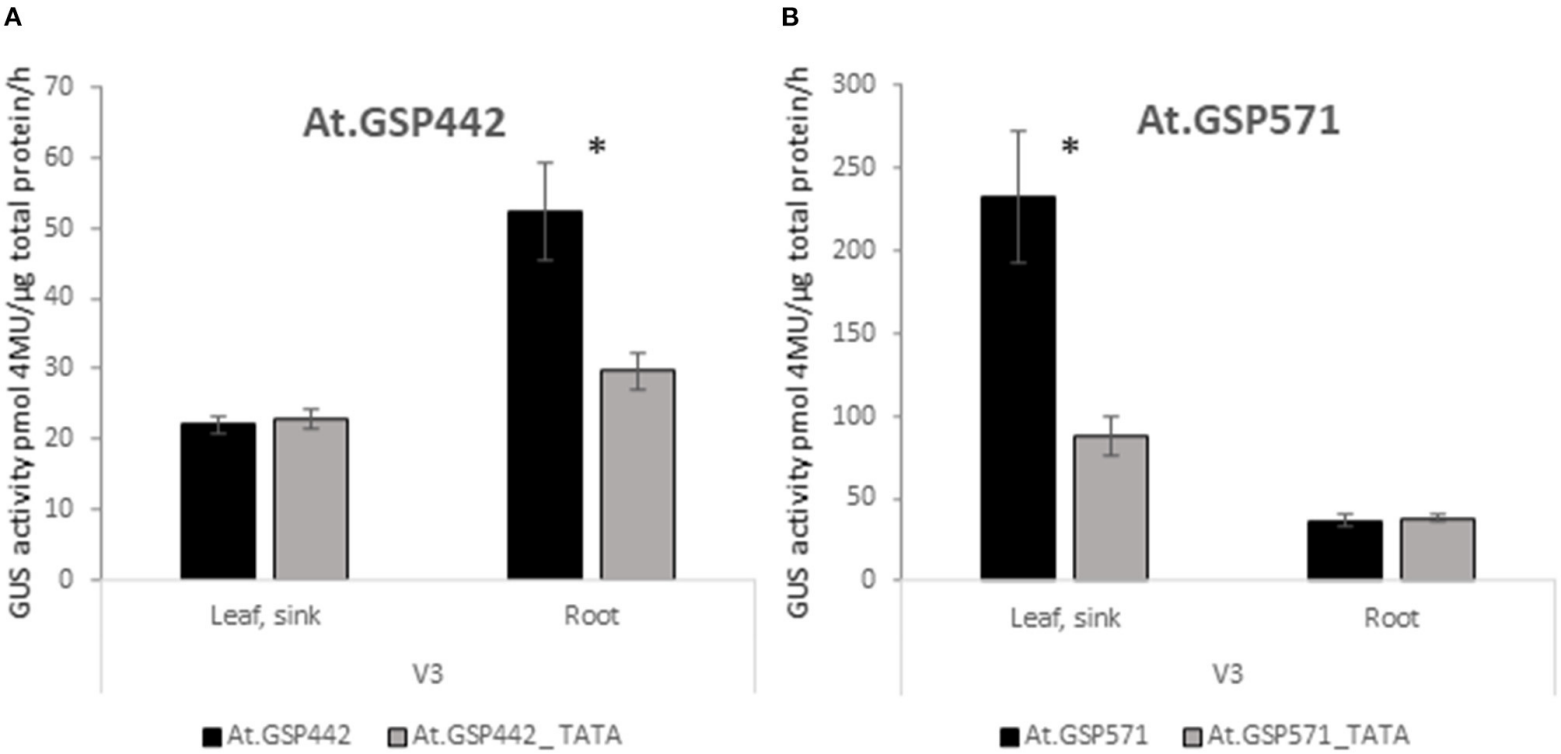
GSPs require the TATA box for function. Expression of GUS reporter genes with TATA box variants of GSPs was compared to original At.GSP442 **(A)** and At.GSP571 **(B)** promoters. At.GSP442_TATA and At.GSP571_TATA promoter variants were generated by introducing transversions in the TATA boxes of the respective promoters. Promoter variants were tested in the same expression cassette context as the original promoters in Figure 1. GUS reporter gene activity was performed on leaf and root tissues from transgenic soybean plants at the V3 stage. GUS enzyme activity on MUG substrate was normalized to total protein and reported as pmol 4MU/μg total protein/h. At least 12 independent transgenic events were analyzed, and the data are reported as the mean with SE. *Indicates a statistically significant difference between original and TATA variants when compared in the same tissue type with *p*-value threshold corrections to control for false discovery <0.05 in multiple testing.

To test whether the TATA box is necessary for promoter-driven expression in these promoters, mutant variants of both At.GSP442 and At.GSP571 were generated by introducing transversions in the intended TATA box motif to replace the TATA box sequence with GC-rich sequences. The resulting TATA box variants were tested in the context of the same reference expression cassette as the original promoters to compare the GUS activity in the variants to the original versions of the respective promoters. Each variant showed significant decreases compared with the original promoters (*p* < 0.025; Figures 4A,B). The At.GSP442_TATA variant GUS activity in the V3 root was 43% lower than the original promoter and was reduced to near the assay quantification limit similar to the expression in the V3 leaf. The At.GSP571_TATA variant GUS activity in V3 leaf decreased by 72% but still maintained a low level of activity, suggesting that alternative but less effective transcription initiation sequences may be utilized in the variant. These results indicate that the TATA box is necessary for the appropriate function of these promoters.

### GSIs Can Enhance Expression and Demonstrate Predictable Splicing

**Figure 5:**
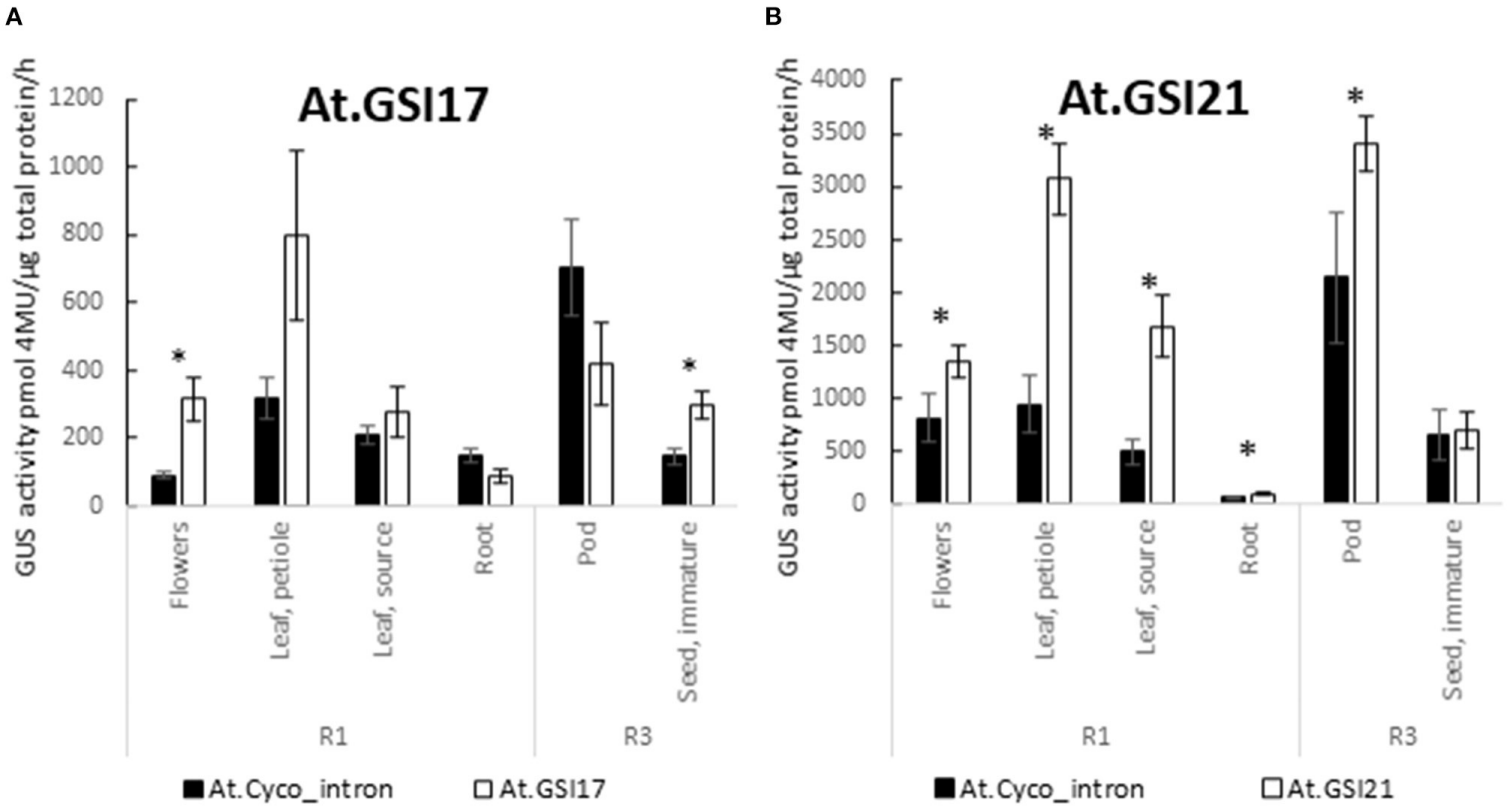
GSIs enable effective expression. Introns At.GSI17 **(A)** and At.GSI21 **(B)** were tested in the context of At.GSP576 and At.GSP571, respectively, and the expression from cassettes with GSIs was compared with At.Cyco_intron when paired with the same promoter. GUS reporter gene activity was performed on leaf, root, flowers, pods, and seeds from transgenic soybean plants at R1 and R3 stages. GUS enzyme activity on MUG substrate was normalized to total protein and reported as pmol 4-MU/μg total protein/h. At least six independent transgenic events were analyzed, and the data are reported as the mean with standard error. *Indicates a statistically significant difference between At.Cyco_intron and the GSI when compared in the same tissue-type with *p*-value threshold corrections to control for false discovery <0.05 in multiple testing.

Building on the GSP cassettes, GSIs were substituted for *At.Cyco*_intron within the 5′ UTR of the expression test cassettes and were evaluated for their ability to drive effective expression and direct effective splicing. At.GSI21 was tested in combination with At.GSP571, and At.GSI17 was tested in combination with At.GSP576 (Table 1, cassettes 7 and 8). Both GSIs in the context of the relevant GSP cassette produced GUS expression levels at least comparable to those observed when using the *At.Cyco*_intron (Table 1, cassettes 3 and 4) and further enhanced expression in some tissues (Figure 5). At.GSI21 showed a significant enhancement of expression relative to the comparable At.GSP571 cassette with *At.Cyco*_intron in R1 flowers, leaf petiole, leaf source, root, and R3 pod (*p* < 0.042). At.GSI17 modified the expression profile of the comparable At.GSP576 cassette with At.Cyco_intron, with significant enhancement of expression, observed in R1 flowers and R3 seed (*p* < 0.025). The expression also appeared to be increased in leaf petiole and reduced in a pod but was not statistically significant.

To test whether the key molecular function of the introns followed predictions, transcript characterization was conducted to elucidate splicing patterns in *At.Cyco*_intron, At.GSI17, and At.GSI21. Detailed molecular characterization was performed by generating cDNA libraries from leaf RNA by reverse transcription, followed by sequencing library generation for high throughput sequencing. Sequencing reads were trimmed and mapped to the expression cassette sequences to obtain both sequence-specific information on splice junctions, as well as quantitative information on the frequency of splice site usage (Table 2). For each cassette, a minimum of 10,000 reads that uniquely mapped to the expression cassette were generated (Supplementary Material S4). Mapped reads were further analyzed to identify splice junctions across the expression cassette. Reads that demonstrated expected splicing at the predicted 5′ and 3′ sites, unspliced transcripts at the predicted 5′ and 3′ sites, as well as any unexpected splice junctions were quantified (Table 2). Splice junctions observed for the 5′ UTR and *St.LS1* introns indicate that the intended splice sites enable efficient splicing. To assess the predictability of the splicing at the intended 5′ and 3′ sites, read counts mapped to each of the intended splicing nucleotide positions were categorized as expected splicing, unspliced, or unexpected splicing, and the resulting number of reads in each category was normalized to the total number of reads mapped to the position. The resulting usage efficiency of the intended splicing position was reported as % observed at the expected 5′ or 3′ splicing position. The majority of mapped transcripts demonstrated expected splicing, with expected splice site usage ranging from 82.6 to 98.9% across both 5′ and 3′ splicing nucleotide positions for all three cassettes. Minor amounts of unspliced introns and alternative splicing were detected for all three 5′ UTR introns. For the *At.Cyco*_intron, 1.2 and 1.5% unspliced reads were detected at the intended 5′ and 3′ splice sites, respectively. In At.GSI17, 2.8 and 3.2% of reads at the 5′ and 3′ splice sites were unspliced, respectively, and 4.2 % of reads at the 5′ splicing nucleotide positions were found to be spliced to an alternative 3′ site 13 bases downstream of the intron. In At.GSI21, reads that mapped to the intended 5′ and 3′ splice sites were found to be unspliced in 10.7 and 10.8% of reads, respectively, and alternative splicing was observed in 6.6% of reads at both positions. Two alternative 5′ splice sites and one alternative 3′ splice site were detected in these alternatively spliced reads. These results are within the range of intron-splicing efficiencies reported in Arabidopsis and soybean studies, where detectable levels of alternative splicing (including intron retention and alternative 5′ and 3′ splice sites) are found in the majority of Arabidopsis and soybean genes (Lorkovic et al., 2000; Filichkin et al., 2010; Marquez et al., 2012; Iñiguez et al., 2017; Chaudhary et al., 2019; Song et al., 2020). In summary, the GSIs demonstrated predictable splicing, with all detected splice junctions in the reporter gene found to be using at least one of the intended splice sites. Overall > 82% of the reads at the intended splice sites of GSIs were spliced as expected.

**Table 2:** GrassRootsIntrons (GSIs) demonstrate predictable splicing that is dependent on functional splicing motifs.

**Intron description**	**5^**′**^ splice site**	**3 ^**′**^ splice site**	**Splicing category**	**Trimmed read count**	**% reads obs at expected 5^**′**^ splice site**	**% reads obs at expected 3^**′**^ splice site**
At.Cyco	511	853	Expected splicing	5,432	98.8	98.5
At.Cyco	511	853	Unspliced	65 (5**′**), 80 (3**′**)	1.2	1.5
At.GSI17	511	805	Expected splicing	52,618	93.1	96.7
At.GSI17	511	805	Unspliced	1,599 (5**′**), 1,809 (3**′**)	2.8	3.3
*At.GSI17*	*511*	*818*	*Unexpected splicing*	*2,304*	*4.1*	*-*
At.GSI17_IME	511	805	Expected splicing	37,254	93.1	96.8
At.GSI17_IME	511	805	Unspliced	1,070 (5**′**), 1,241 (3**′**)	2.7	3.2
*At.GSI17_IME*	*511*	*818*	*Unexpected splicing*	*1,674*	*4.2*	*-*
At.GSI17_splicesite	511	805	Expected splicing	0	0	0
At.GSI17_splicesite	511	805	Unspliced	28,436 (5**′**), 20,394 (3**′**)	100	100
At.GSI21	511	814	Expected splicing	17,324	82.6	83.5
At.GSI21	511	814	Unspliced	2,269 (5**′**), 2,212 (3**′**)	10.8	10.7
*At.GSI21*	*511*	*673*	*Unexpected splicing*	*1,385*	*6.6*	*-*
*At.GSI21*	*688*	*814*	*Unexpected splicing*	*187*	*-*	*0.9*
*At.GSI21*	*723*	*814*	*Unexpected splicing*	*1,036*	*-*	*5.7*
At.GSI21_IME	511	814	Expected splicing	14,207	79.7	80.9
At.GSI21_IME	511	814	Unspliced	2,570 (5**′**), 2,626 (3**′**)	14.4	15.0
*At.GSI21_IME*	*511*	*673*	*Unexpected splicing*	*1,047*	*5.9*	*-*
*At.GSI21_IME*	*688*	*814*	*Unexpected splicing*	*2*	*-*	*0.0*
*At.GSI21_IME*	*723*	*814*	*Unexpected splicing*	*724*	*-*	*4.1*
At.GSI21_splicesite	511	814	Expected splicing	0	0	0
At.GSI21_splicesite	511	814	Unspliced	6,746 (5′), 5,768 (3′)	100	100
*At.GSI21_splicesite*	*580*	*673*	*Unexpected splicing*	*704*	*-*	*-*
*At.GSI21_splicesite*	*580*	*827*	*Unexpected splicing*	*165*	*-*	*-*
*At.GSI21_splicesite*	*633*	*827*	*Unexpected splicing*	*146*	*-*	*-*
*At.GSI21_splicesite*	*723*	*827*	*Unexpected splicing*	*716*	*-*	*-*

*Rows with italic values have unexpected splicing*.

**Figure 6:**
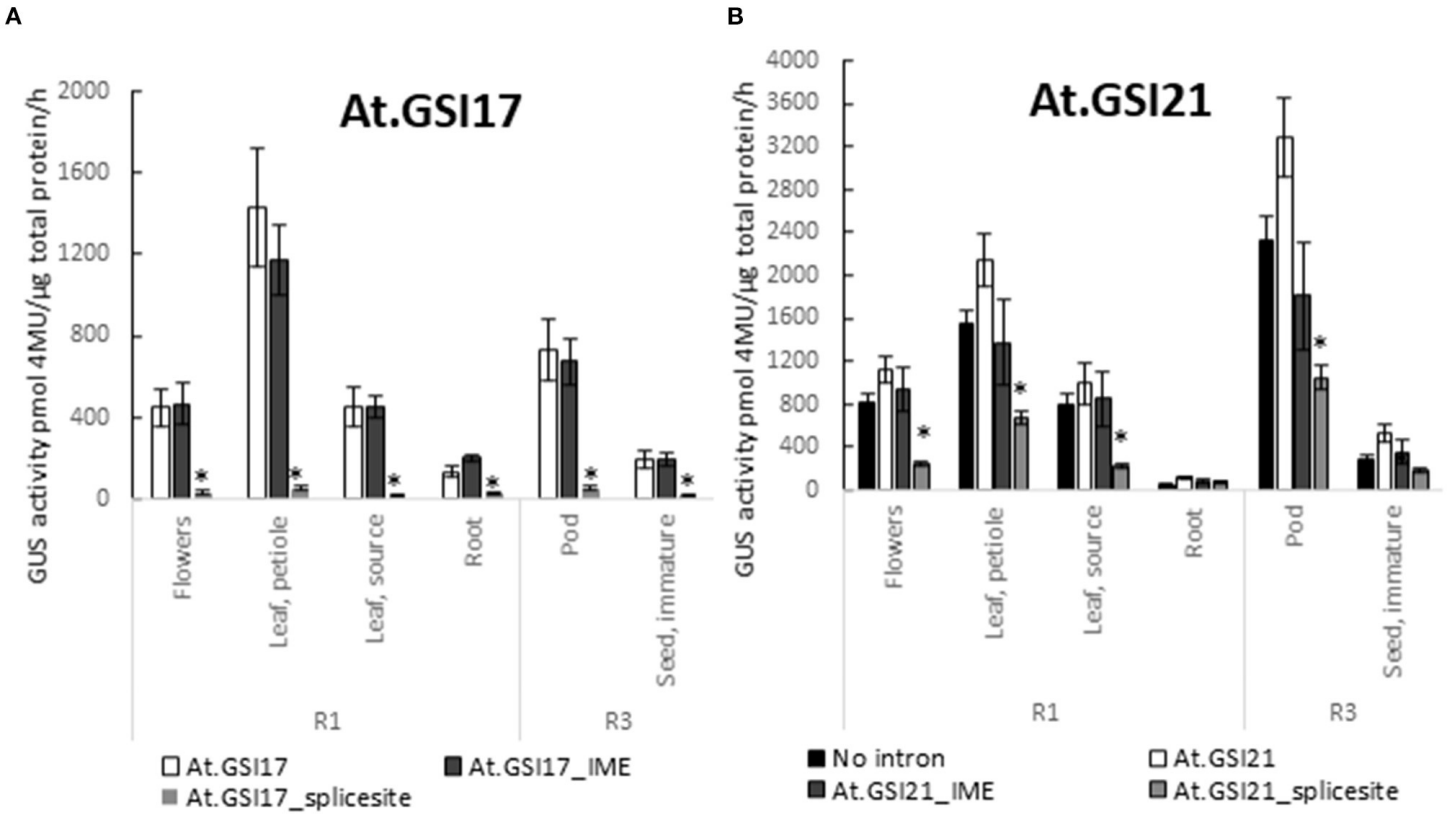
GSIs require intron motifs for function. Expression of GUS reporter genes in the IME motif and splice site variants of GSIs was compared with the original At.GSI17 **(A)** and At.GSI21 **(B)** introns. At.GSI17_IME and At.GSI21_IME intron variants were generated by introducing transversions in the IME motifs and splicing motifs in the respective intron sequences (Supplementary Material S1). Intron variants were tested in the same context as the original introns as in Figure 5. GUS reporter gene activity was performed on various tissues from transgenic soybean plants at R1 and R3 stages. GUS enzyme activity on MUG substrate was normalized to total protein and reported as pmol 4-MU/μg total protein/h. At least six independent transgenic events were analyzed, and the data are reported as the mean with standard error. *Indicates a statistically significant difference between original and mutant variants when compared in the same tissue type with *p*-value threshold corrections to control for false discovery <0.05 in multiple testing.

To test whether known motifs that are necessary for the function of native introns are also required for GSIs, we generated variants of At.GSI17 and At.GSI21 with transversion mutations in both predicted splice sites (At.GSI17_splice site and At.GSI21_splice site), and also in motifs previously identified to be required for intron-mediated enhancement (IME) of expression (Rose, 2008) (At.GSI17_IME and At.GSI21_IME). The resulting intron variants were tested in the context of the same expression cassettes as above (Table 1, cassettes 9, 10, 12, and 13) and evaluated for both GUS reporter gene expression and splicing. Mutation of the IME motifs in both GSIs reduced GUS activity compared to the original intron in the same tissue type, most notably in pod and leaf petiole for At.GSI21, and in leaf petiole for At.GSI17 (Figure 6); however, neither comparison met statistical significance criteria in multiple testing. At.GSI21_IME retained substantial GUS activity across all tissues assayed that is comparable to GSP571 without intron (Table 1, cassette 11), suggesting that expression enhancement can largely be attributed to the IME motifs (Figure 6B). The IME mutations did not substantially alter the splice site usage, consistent with the idea that these IME motifs are not necessary for splicing (Table 2). In contrast, mutations of both splice sites in At.GSI17 and At.GSI21 essentially abolished splicing at the original splice sites (Table 2). About 100% of reads detected at the original splice sites were unspliced in both At.GSI17_splice site and At.GSI21_splice site cassettes. Both At.GSI17_splice site and At.GSI21_splice site introns are expected to generate transcripts with short upstream open reading frames (ORFs) in the intron that would likely not produce protein. This disruption of splicing and protein expression was consistent with the large reductions in GUS reporter gene expression when compared to the corresponding original GSI across all tissues for At.GSI17 (*p* < 0.05), and across all tissues except root for At.GSI21 (*p* < 0.04) (Figure 6). In the expression cassette with At.GSI17_splice site, GUS activity was reduced to near the limit of quantitation across tissue types. Interestingly, for At.GSI21_splice site, the expression levels were reduced below the no intron control (*p* < 0.04), but the At.GSI21_splice site expression cassette still retained a low level of activity. Consistent with this observation, unexpected splicing was detected in At.GSI21_splice site with increased usage of alternative splice sites (Table 2). One of the alternatively spliced transcripts (5′ splice site at 580 and 3′ splice site at 673) results in an alternative translation start that is upstream and in-frame with the GUS-coding sequence and may explain the low level of GUS protein activity observed. Altogether, these results indicate that effective splicing and function of the GSIs are dependent on the splice sites that determine interactions with spliceosome machinery, whereas the IME motifs contribute to the expression enhancement.

### GSTs Can Drive Effective Expression With Transcript Termination

**Figure 7:**
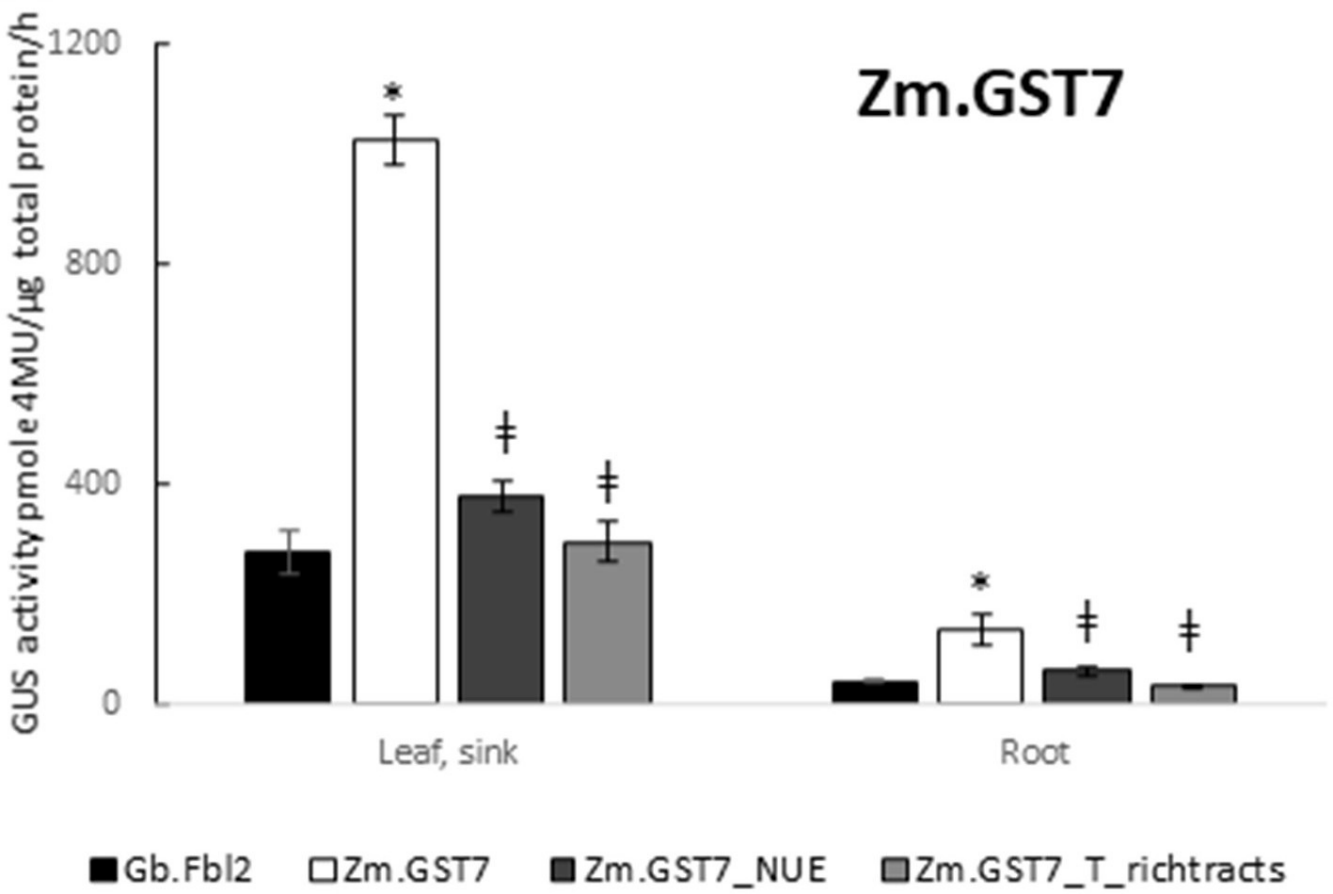
GST7 enables effective expression that is dependent on functional polyadenylation and cleavage signals. Expression of GUS reporter genes with Zm.GST7 and its NUE polyadenylation signal and T-rich tract cleavage site variants were compared with Gb.Fbl_3′ UTR in the same expression cassette context with At.GSP571 and At.Cyco_intron. Zm.GST7_NUE and Zm.GST7_T-rich_tracts 3′ UTR variants were generated by introducing transversions in the relevant motifs. GUS reporter gene activity was performed on leaf and root tissues from transgenic soybean plants at the V3 stage. GUS enzyme activity on MUG substrate was normalized to total protein and reported as pmol 4-MU/μg total protein/h. At least 12 independent transgenic events were analyzed, and the data are reported as the mean with standard error. *Indicates a statistically significant difference between Zm.GST7 and Gb.Fbl2, whereas ≠ indicates a statistically significant difference between original Zm.GST7 and mutant variant when compared in the same tissue type with *p*-value threshold corrections to control for false discovery <0.05 in multiple testing.

To test for *in planta* function, Zm.GST7 was substituted for Gb.Fbl2_3′ UTR in the context of the At.GSP571 promoter testing cassette (Table 1, cassettes 3, 14, 15, and 16), and the resulting cassette was transformed into soybean. Compared with the At.GSP571 cassette with Gb.Fbl2_3′ UTR, the cassette with Zm.GST7 showed significantly enhanced expression in both V3 leaf and root (*p* < 0.05; Figure 7). We also tested the expression activity of Zm.GST7 and another computationally derived 3′ UTR, Zm.GST43, in maize leaf protoplasts and found that both GSTs showed high-expression activity (Supplementary Material S5). Furthermore, Zm.GST43 demonstrated effective expression in stably transformed maize (Supplementary Material S5). These results show that GSTs can drive effective gene expression *in planta*.

**Figure 8:**
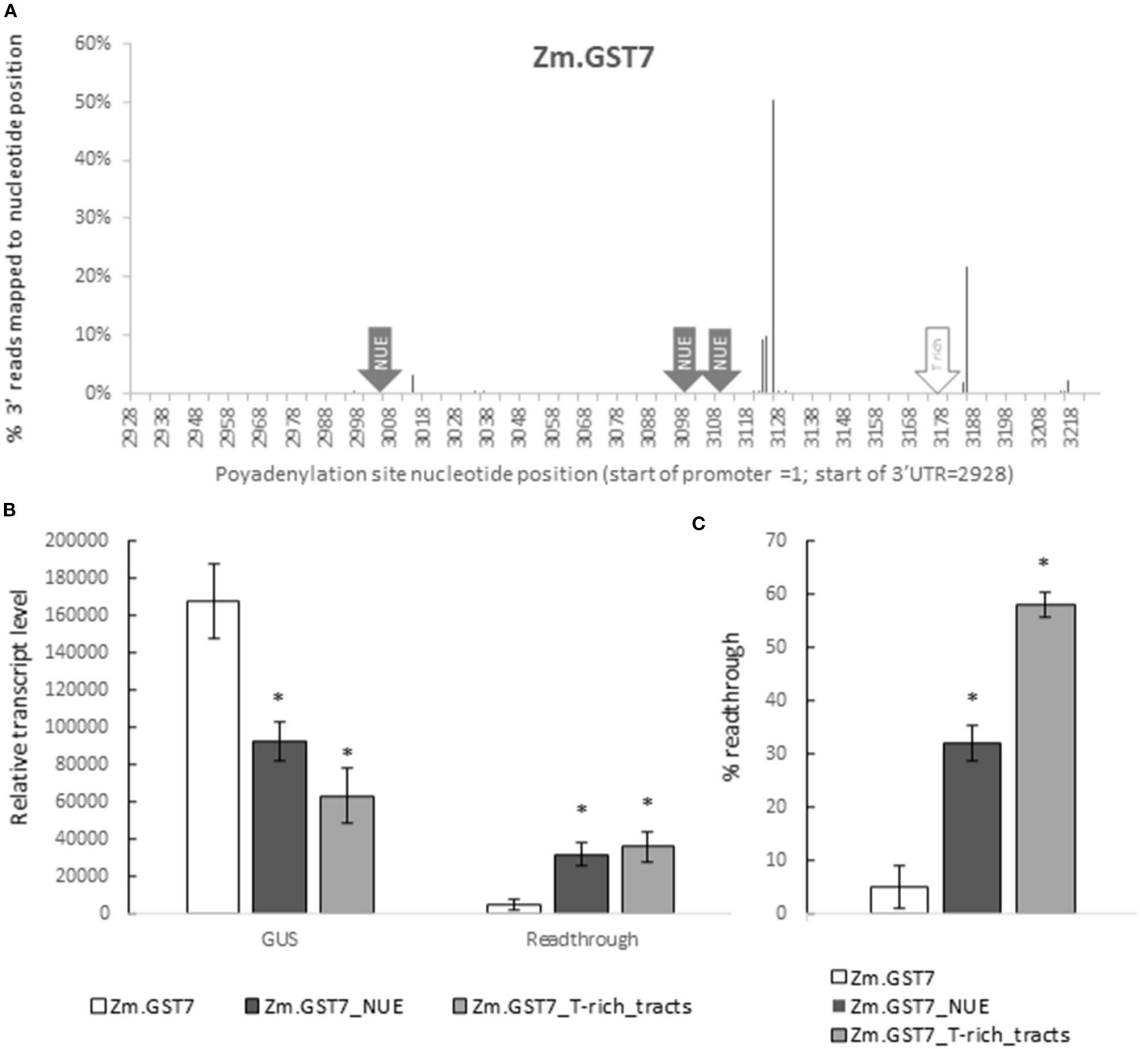
GST7 enables effective transcript termination as predicted. This function is dependent on functional polyadenylation and cleavage signals. 3′ polyadenylation sites for GUS reporter gene with Zm.GST7 were characterized by sequencing **(A)**. 3′ ends of GUS transcript fragments from leaf RNA were captured and sequenced by high throughput sequencing. About 12 independent transgenic events were characterized to produce 479,738 reads mapped to the expression cassette after trimming. About 3,219 of these mapped reads with the 3′ sequencing adapter were identified as polyadenylation sites. The resulting sequencing reads were pooled to report the polyA site distribution as % reads mapped to each nucleotide position. Nucleotide positions are reported with the 5′ most position of the promoter = 1.GUS transcripts and read through from reporter genes with Zm.GST7 and variants in the NUE polyadenylation signal and T-rich tract cleavage site signals were analyzed by qRT-PCR **(B,C)**. GUS and read**-**through transcripts were detected by primer/probe sets in the GUS-coding sequence and downstream of the 3′ UTR, respectively. Transcript levels within each tissue are normalized by two [-DeltaDeltaC(T)] methods and reported as a relative expression value. At least 12 independent transgenic events were analyzed, and the data are reported as the mean with standard error. Percent read through is calculated by normalizing read**-**through transcript to GUS transcript **(C)**. *Indicates a statistically significant difference between original and mutant variants with corrections to control for false discovery in multiple testing.

Zm.GST7 was generated with two polyadenylation sites, similar to the known polyadenylation pattern in effective 3′ UTRs used in current commercialized plant biotech traits, such as the Nopaline synthase 3′ UTR from *Agrobacterium tumefaciens* (Depicker et al., 1982). To assess whether the molecular function of Zm.GST7 followed predictions for transcript polyadenylation, leaf RNA of soybean plants transformed with the Zm.GST7 cassette was analyzed by high-throughput sequencing of 3′ RACE libraries. The resulting sequence reads were mapped to the cassette sequence to identify polyadenylation sites. A total of 479,738 trimmed reads were mapped to the expression cassette. Of those, 3,219 reads aligned with the 3′ sequencing adaptor to define polyadenylation sites. As expected, two dominant and concentrated polyadenylation sites were found, centered around nucleotide positions 3,126 and 3,186 (Figure 8A). Nucleotide 3,126 is an A in a YA dinucleotide within a T-rich region that is downstream of 2 AATAAA consensus sites, the closer being 17 bp away, which is one of the expected configurations of polyadenylation sites (Li and Hunt, 1995). Nucleotide 3,186 is positioned at the end of a poly Tract, which has also been found to be enriched near cleavage sites (Wu et al., 2011). Overall, the polyadenylation sites were found to be consistent with plant native 3′ UTRs and other 3′ UTRs commonly used in plant biotechnology.

To determine if Zm.GST7 enabled effective transcript processing, qRT-PCR assays were designed to quantify transcript read through. This assay is comprised of two DNA primer-probe sets that enabled quantification of the relative ratio between transcripts detected within the GUS-coding region and transcripts detected downstream of the 3′ UTR. *Gb.Fbl2*_3′ UTR has typically shown a read through of 17–20% when used in a soy transgene (data not shown). For Zm.GST7, read through was detected at <5% of the GUS-coding sequence (Figures 8B,C). In maize, Zm.GST43 read through was very low, near the limit of quantitation (Supplementary Material S5). These results indicate that the transcripts processed on GSTs are predictable and have minimal read through to downstream sequences. Therefore, computationally derived 3′ UTRs present minimal risk with regard to having an adverse impact on neighboring transgenes in a trait stack.

To further assess the role of known motifs for polyadenylation such as the NUE or the T-rich tracts, variants of Zm.GST7 were generated with these motifs disrupted. The resulting Zm.GST7 variants, Zm.GST7_NUE, and Zm.GST7_T-rich_tracts were introduced into the same expression cassette in soybean to substitute for the original Zm.GST7. The two Zm.GST7 variants were then compared with the original in terms of both expression and molecular function. Both Zm.GST7_NUE and Zm.GST7_T_rich tracts cassettes were found to have significantly reduced expression of the GUS reporter as compared with Zm.GST7 in both leaf and root (*p* < 0.05) by >50% (Figure 7). GUS transcript analysis from leaf tissue corroborated the GUS enzymatic assay, with transcript reductions in GUS-coding sequence of the variants as compared with the original Zm.GST7 (*p* < 0.05; Figures 8B,C). As expected, increased levels of read-through transcripts were detected in both Zm.GST7 variants, as compared with the original Zm.GST7 (*p* < 0.05), resulting in an overall increase in percentage read through, with a 6-fold increase in the read through observed in Zm.GST7_NUE and a >10-fold increase in Zm.GST7_T-rich_tracts. Interestingly, while the mutations in Zm.GST7_NUE reduced expression and increased read through, the overall distribution of detected polyadenylation transcripts that mapped within the cassette was still similar to Zm.GST7, whereas, for Zm.GST7_T-rich_tracts, no polyadenylation transcripts were mapped within the 3′ UTR (Supplementary Material S6). These results indicate that, while the NUE contributes to the efficiency of 3′ UTR processing and expression, the T-rich tracts are required for defining the cleavage site for polyadenylation.

Finally, to demonstrate that these computationally derived expression elements can function together in an expression cassette, the plant sourced promoter and leader, intron, and 3′ UTR were fully substituted for At.GSP571, At.GSI21, and Zm.GST7, respectively (Supplementary Material S7). The expression was compared to the well-known CaMV.35S promoter as a reference. The data showed that the computationally derived expression elements were compatible and together, were able to drive leaf expression in the vegetative stage, which are comparable to 35S with intron enhancement. The results indicate that these new expression elements can be used not only to replace individual elements; they can also be used to generate new and unique expression cassette combinations that can further expand the opportunities for optimizing transgenic traits.

## Discussion

The availability of diversified expression elements for efficacious and predictable gene expression regulation is one of the key challenges in developing new plant biotechnology traits to meet the growing demands of farmers and consumers. In this study, we have generated a set of new and functional gene expression elements by using computational methods to learn from native sequences sourced from co-expressed plant genes. Our results demonstrate that these new expression elements can drive effective expression of a transgene and perform with the molecular characteristics of native expression elements. In all cases, we have found that overall expression levels from cassettes with these computationally derived elements were at least comparable to the reference cassette with plant native expression elements across multiple tissues and developmental stages. The new expression elements all contributed to the unique expression profile and levels of the transgene. The specific expression levels and profiles conferred by the promoters could be further modified by introns and 3′ UTRs. By developing new promoters, introns, and 3′ UTRs, we have generated a modular and diversified expression tool kit for optimizing plant biotechnology traits. Moreover, these computationally derived expression elements have no significant sequence identity to the source genome of the training set, thus offering the additional potential for sequence diversification, while recapitulating the intended molecular functions.

The computationally derived expression elements demonstrated molecular functions that are consistent with well-understood mechanisms in native plant genes. For example, transcription start sites from GSPs were detected ~30 bp downstream of the AT-rich TATA box and occurred near Inr-like motifs, as observed in genome-wide analysis of plant transcription (Hetzel et al., 2016). The majority of transcripts from expression cassettes with GSIs spliced as expected, with a minor amount of intron retention and alternative splicing observed. This is consistent with reports that up to 70% of genes in plants, including Arabidopsis and soybean, are alternatively spliced (Lorkovic et al., 2000; Filichkin et al., 2010; Marquez et al., 2012; Iñiguez et al., 2017; Chaudhary et al., 2019). GSTs enabled transcript processing and termination with low- to no-read-through downstream of the 3′ UTR. For each of the GSTs, two polyadenylation sites were observed near known motifs in native plant 3′ UTRs. These motifs include the AU-rich Near Upstream Element (NUE) upstream of the polyadenylation site and the T-rich tract near the cleavage site (Li and Hunt, 1995; Wu et al., 2011). The presence of two polyadenylation sites was also consistent with the use of multiple polyadenylation sites in ~70% of genes in plants (Shen et al., 2008; Wu et al., 2011). Mutations of key motifs associated with these molecular functions in native sequences, including the promoter TATA box, intron splice sites, and 3′ UTR NUE, and T-rich tracts resulted in reduced expression levels, indicating that these key motifs are necessary for driving expression through interactions with the cellular machinery. These data, together, support the idea that the computationally derived expression elements leverage the same cellular and molecular mechanisms as native expression elements to recapitulate these molecular properties.

In addition, the new expression elements have demonstrated desirable properties for optimizing plant biotechnology transgenes. For example, the GSPs demonstrated a dominant transcriptional start site (TSS) peak that is predictably positioned ~30 bp downstream of the intended TATA box. Some native plant and animal promoters have been observed with a dominant TSS peak, but others have a broad transcription start region spread across the promoter and 5′ UTR (Morton et al., 2014; Mejia-Guerra et al., 2015). While both of these TSS profiles can drive transgene expression, as we have observed with *At. Cyco*_promoter and GSPs, a broad start region has some drawbacks for biotechnology. First, in angiosperms, small open-reading frames upstream of the main coding sequence are common but can decrease protein expression (von Arnim et al., 2014). Second, the scanning model for translation favors initiation at the first ATG codon encountered by the ribosome (Kozak, 1989a,b). Therefore, optimized promoters with a dominant transcription start site and 5′UTRs that are devoid of ATGs can enable a more predictable translation of the transgene-coding sequence.

Similarly, the GSIs and GSTs present opportunities for enhancing the predictability of transgene expression. For example, alternative splicing has been widely reported in native plant genes, including intron retention, exon skipping, and the use of alternative splice sites, and has been proposed to be a mechanism for tissue or development-specific gene regulation, or response to environmental conditions (Lorkovic et al., 2000; Filichkin et al., 2010; Marquez et al., 2012; Iñiguez et al., 2017; Chaudhary et al., 2019; Song et al., 2020; Martín et al., 2021). The GSIs characterized in this study demonstrated mostly predictable splicing and did not disrupt the GUS-coding sequence, indicating that this is a potential way to reduce expression variability. While our current data demonstrate predictable splicing in one tissue, our computational approach has the potential for learning from new genomic datasets to further optimize introns for predictable splicing across tissues and conditions. Recent improvements in molecular characterization and sequencing of transcripts (Steijger et al., 2013; Wang et al., 2016) will further improve the resolution of training sets to enable optimization of expression predictability and specificity of these elements.

GrassRoots Terminators provide another example of how computationally derived elements can be used to increase the predictability of transgene expression. Native plant genes utilize a variety of alternative polyadenylation sites, which can sometimes produce alternative coding sequences (Wu et al., 2011). The GST characterized in this study demonstrated just two polyadenylation sites as intended, and with little to no detectable read through into downstream sequences. Hence, a combination of optimized 3′ UTRs, in addition to codon optimization of the transgene to avoid unintended polyadenylation, can enable the production of transcripts with predictable coding sequences.

While our results have demonstrated predictability of native expression element motifs in these new expression elements, our results also show that additional mechanisms are providing function outside of these identified motifs. For example, disrupting the TATA box significantly reduced but did not fully abolish expression. IME motifs that were tested could only partially explain the intron-enhancing effect on expression. Specific mutations of the NUE reduced expression, and disruptions of T-rich regions increased read through, but neither set of mutations completely abolished expression. These results suggest that the computationally derived expression elements contain additional motifs within the respective expression elements that provide function in the variants. Alternative motifs for transcription initiation (Morton et al., 2014; Mejia-Guerra et al., 2015; Hetzel et al., 2016), IME (Parra et al., 2011), polyadenylation, and cleavage (Wu et al., 2011) have been reported. Recent reports of expression elements generated by combinatorial approaches with discreet modular sequence fragments or motifs have demonstrated enhanced function in plants (Liu et al., 2014; Sahoo et al., 2014; Rushton, 2016; Belcher et al., 2020). With a computational approach, our expression elements are the result of learning from the analysis of a larger set of native sequences, in which the generated sequences are predicted to contain combinations of motifs that can potentially enhance the robustness of expression. Further advances in genomic datasets and learning algorithms can enhance motif discovery and lead to further improvement in robustness of expression element performance.

Expression elements derived from larger sequence training sets from co-expressed genes may drive gene expression profiles with increased robustness and predictability. For example, At.Cyco has previously been identified in the Arabidopsis expression Atlas (Schmid et al., 2005; Klepikova et al., 2016) as a broadly expressed gene with an annotated TSS (Swarbreck et al., 2008). Interestingly, when the native promoter from this plant gene was utilized to drive the expression of a GUS reporter transgene, we found that the resulting expression profile was developmentally regulated. This indicates that transgene expression profiles do not always recapitulate those observed in nature when using native expression elements and could change depending on the gene expression cassette composition and/or genomic context, which underscores the importance of testing and characterization in the transgenic context. Interestingly, the characterized GSPs in this study generally recapitulated the overall expression pattern preferences of the training set. While our current data only showcase three promoter examples from two different training sets, this highlights the opportunity for using this computational approach to optimize tissue and developmental-stage specific expression of plant biotechnology traits.

The focus of this research is to optimize expression elements to recapitulate properties of native elements and drive efficacious expression of transgenes. Although our work features computationally derived expression elements, it does not constitute the synthetic biology of plants. In microbes, synthetic biology has assembled entire synthetic metabolic pathways of sequentially acting enzymes, or genetic circuits of interacting regulatory factors (Patron et al., 2015; Shih et al., 2016; McCarthy and Medford, 2020; Sorg et al., 2020). The expression elements described in this study are DNA sequences that can be used to drive the expression of plant biotechnology traits, but in and of themselves are not intended to be expressed as enzymes or regulatory components. While these new expression elements can be useful for metabolic engineering applications, they only serve the same roles that native elements have in previously developed, risk assessed, and commercialized plant biotechnology traits to deliver functional expression of transgenes.

Overall, we have generated functional expression elements by learning from native plant sequences and expression data. We have generated new elements by using sequence training sets from multiple plant species (Arabidopsis and maize) to be applied to different target crops (soybean and maize), and we have shown that these design principles can be applied to different types of expression elements (promoters, 5′ UTRs, introns, and 3′ UTRs). Furthermore, these different expression elements can be used together or with native elements to generate unique and optimized combinations for the expression of agronomic trait genes. Applications of this technology can be expanded to other crops and element functions to further optimize and diversify expression elements for developing future plant biotechnology traits.

## Data Availability Statement

The original contributions presented in the study are included in the article/Supplementary Material, further inquiries can be directed to the corresponding author.

## Author Contributions

ID, JT, MM, and AS conceived and designed the experiments. JT and TE supervised the execution of research. CB, KD, RG, ZG, OH, JJ, HL, MM, BO'B, AS, and JT generated the data and contributed to the analysis. JT assembled the figures and wrote the manuscript. All authors contributed to the article and approved the submitted version.

## Funding

Funding for this research was provided by NSF small business grants to GrassRoots Biotechnology on constitutive promoters for crop improvement: NSF 0810649 (STTR Phase I), NSF 0957836 (STTR Phase II), and NSF 0957836 (STTR Phase IIB), as well as resources from GrassRoots Biotechnology, Monsanto Company, and Bayer Crop Science.

## Conflict of Interest

The research activities in this report were conducted by teams at GrassRoots Biotechnology, Monsanto Company, and Bayer Crop Science. The authors contributed to this research as employees of one or more of the above entities. ID and AS are inventors of the new plant regulatory elements on a granted patent assigned to Monsanto Company. The remaining authors declare that the research was conducted in the absence of any commercial or financial relationships that could be construed as a potential conflict of interest.

## Publisher's Note

All claims expressed in this article are solely those of the authors and do not necessarily represent those of their affiliated organizations, or those of the publisher, the editors and the reviewers. Any product that may be evaluated in this article, or claim that may be made by its manufacturer, is not guaranteed or endorsed by the publisher.
