# Supplementary material for: Expression Elements Derived From Plant Sequences Provide Effective Gene Expression Regulation and New Opportunities for Plant Biotechnology Traits
Source: Front Plant Sci. 2021 Oct 22;12:712179. doi: 10.3389/fpls.2021.712179 (PMC8569612; doi:10.3389/fpls.2021.712179)
Supplement: Supplementary file 1 [file Data_Sheet_1.pdf]

S1 Sequence lists

>At.GSP442

TGTTAATGTTATCCGAACTAGTCATAATTACAACCGACAAAATAAGGTTATTTTGTGTGTTATA
GAATTTTTTGGACAGTTTTTGTTTTGGTTTTCGATTGTAGTAAAAATAGATTTATGTAATAAGA
TTTACTTTTCTTGTTGAAACAAAATAATCTTAGAATTAACTCAACTTTTATGTTAGAACAAATG
ATAAAAAAATTTCCCCTTTTCTATGCGATTATTTTCAATCAGAGAGAAATACATATAATATATA
TAATTCAAATTAATCTGCCAAATTAATAAATTTGGATTAAAATTTATAAATGAAACAATGGTGT
AAGGCAATTAAAAACACAACACTAAAAATATGAGAACATTTTATCTGGGCATTAAGAGTTTGGG
CTTTAGATCTAAAATAAAGGCCGGCCCAACGAGAATATTAAACCCTAATTGACCTAGTTCCCTA
TATATATAAACCCTATATTTCTCTCGTCACTCCTCAACTCTCAGCTAAACCA

>At.GSP442_TATA

TGTTAATGTTATCCGAACTAGTCATAATTACAACCGACAAAATAAGGTTATTTTGTGTGTTATA
GAATTTTTTGGACAGTTTTTGTTTTGGTTTTCGATTGTAGTAAAAATAGATTTATGTAATAAGA
TTTACTTTTCTTGTTGAAACAAAATAATCTTAGAATTAACTCAACTTTTATGTTAGAACAAATG
ATAAAAAAATTTCCCCTTTTCTATGCGATTATTTTCAATCAGAGAGAAATACATATAATATATA
TAATTCAAATTAATCTGCCAAATTAATAAATTTGGATTAAAATTTATAAATGAAACAATGGTGT
AAGGCAATTAAAAACACAACACTAAAAATATGAGAACATTTTATCTGGGCATTAAGAGTTTGGG
CTTTAGATCTAAAATAAAGGCCGGCCCAACGAGAATATTAAACCCTAATTGACCTAGTTCCCGC
GCGCGCTAAACCCTATATTTCTCTCGTCACTCCTCAACTCTCAGCTAAACCA

>At.GSP571

AGTACAATCATACAAGAGCAATATATATATATTTTTGGTTATTGAAATTTAAATATCATCTTCACA
AAATGAAAAAGCACAAAAAGTATTAATTAATATCATGTTTTGAGACTCCTTTTTACCAAGAATA
TAAATTTCACACCTAAGAAAATTCTGAACTAGGAAAATAACCAGCATACAATTAAGGAATAAGA
AAATGCAATTACGATAAACACTTGTCACAAATTGTTTAATAAGTCACTATCCAATCAATTATCA
AAAGTAAGATATTGCCACGTGGCAACCAGTATTTTCATCACCTTATCAAAAGATAAGCAAAAGA
ACCACATCAAAGCCACAAAATGCCAACCACAGATGGATAAGGAAAATCCAACCAACCACATGTA
ATCCCACACCTCATCACCTTATCCACACCTCTGTCTATATATATAAACACACACTTCGTAACCA
CTCATCACTCACCACAAACAGAGAATATCTCATCTCTTCTTAGCAAACAAAG

>At.GSP571_TATA

AGTACAATCATACAAGAGCAATATATATATATTTTTGGTTATTGAAATTTAAATATCATCTTCACA
AAATGAAAAAGCACAAAAAGTATTAATTAATATCATGTTTTGAGACTCCTTTTTACCAAGAATA
TAAATTTCACACCTAAGAAAATTCTGAACTAGGAAAATAACCAGCATACAATTAAGGAATAAGA
AAATGCAATTACGATAAACACTTGTCACAAATTGTTTAATAAGTCACTATCCAATCAATTATCA
AAAGTAAGATATTGCCACGTGGCAACCAGTATTTTCATCACCTTATCAAAAGATAAGCAAAAGA
ACCACATCAAAGCCACAAAATGCCAACCACAGATGGATAAGGAAAATCCAACCAACCACATGTA
ATCCCACACCTCATCACCTTATCCACACCTCTGTCGCGCGCGCTAAACACACACTTCGTAACCA
CTCATCACTCACCACAAACAGAGAATATCTCATCTCTTCTTAGCAAACAAAG

>At.GSP576

AATTAAATTCAACACGTTTGTTATATATTTTTTATTGAAATTATTCTTCATTCGTCTTTTAATG
GATAAAAAGGTATAATCAAGTATATTTTATACACATCTTTCTATTTGTGTGTACCAAATGTTAA
AATGGCCAATTTTGACCAAAAAACCGCATAATTTTCTTAATTTCTTAAATATGATTAATTCATC
AATAACTTGGAATTTCACAATACACAAAAGTGGGTGTAGTTACCGTTATTATATTTATACACAA
CAACTCATCTCCTCATAGAAAGAAAAGAAAAATAAAATAAGAAATCAAAAAACGACAAGATAAC
CAATCTCCACATCATCCACGTGGCGTAAGGATAAGGTCACAACCACCACTCAGCCACGTGGCAG
AATCTTATCCAATCACTCTCACCACACAAACCTTATCCACTTCTATATATAATCTCTTCTTCTC
ATTATCACTCACCACACATCCTTGCAAAAGTAAAGAGAAAAAACAAACAAGA

>At.GSI17

CAGGTAAACCCAGATCTCTTTCTTCTCTTCTCTTCATCTCGATCTCTCCATTTTCATAAACCCA
ATTTTTTCTCTGATTTGTTTGATTTGGTTTGGATCTTTCTGTGTTTCCATGGTTTTAGGAATTT
TAGGATAGATTTTTGTTTGTTCATGTTATTCATCGGATATATAGATTTCAAATTCTTTTGCAAT
TTTTCTCTCTCTTTAGTTTTGCTCAATTTTGGTTGTTGTTGTGATGAGTGTTCTCTTTATGGGT
TTATCTGAGCTTGGTGAGAGTTTTTTGATATTGATTTTGCAGGT

>At.GSI17_IME

CAGGTAAACCCAGATCTCTTTCTTCTCTTCTCTTCATCGATCGAGCTCCATTTTCATAAACCCA
ATTTTTTCTCTGATTTGGGGTCGGGTGTTTGGATCTTTCTGTGTTTCCATGGTTTTAGGAATTT
TAGGATAGATTTTTGTTTGTTCATGTTATTCATCGGATATATAGATTTCAAATTCTTTTGCAAT
TTTTCTCTCTCTTTAGTTTTGCTCAATTTTGGTTGTTGTTGTGATGAGTGTTCTCTTTATGGGT
TTATCTGAGCTTGGTGAGAGTTTTTTGATATTGATTTTGCAGGT

>At.GSI17_ splicesite

CCTTGCCCAACAGATCTCTTTCTTCTCTTCTCTTCATCTCGATCTCTCCATTTTCATAAACCCA
ATTTTTTCTCTGATTTGTTTGATTTGGTTTGGATCTTTCTGTGTTTCCATGGTTTTAGGAATTT
TAGGATAGATTTTTGTTTGTTCATGTTATTCATCGGATATATAGATTTCAAATTCTTTTGCAAT
TTTTCTCTCTCTTTAGTTTTGCTCAATTTTGGTTGTTGTTGTGATGAGTGTTCTCTTTATGGGT
TTATCTGAGCTTGGTGAGAGTTTTTTGATATTGCGGGGTACTGT

>At.GSI21

CAGGTTTATTCTTCTCTCTCTATCCTCTCTCTTCTGATCTCGATTTGTTTTTTTCGAATCGCTC
TACTTCCAGTTAGATTCTTGATTTGAGATTAATTAGATTGATTATTCTAATCGTTTTTTTTGTA
AGCAATTAAGATTTATCTTGTTTTATGTTTTTCTTTAGGTATGACTTGTTATGTATGTCACTGT
TTCAGATCTGATCCTCTCTGTTGGTTTGTGAATTCTCTTGTATTGTTCTAATCACTGTTTCTGA
ATTTGATTCGGGTTTTTATTGAATTCTTTTTATGTGTTTTGGTATTTGCAGGT

>At.GSI21_IME

CAGGTTTATTCTTCTCTCTCTATCCTCTCTCTTCTGATCTATCGGTGTTTTTTTCGAATCGCTC
TACTTCCAGTTAGATTCTTGATTTGAGATTAATTAGATTGATTATTCTAATCGTTTTTTTTGTA
AGCAATTAAGATTTATCTTGTTTTATGTTTTTCTTTAGGTATGACTTGTTATGTATGTCACTGT
TTCATCGAGTATCCTCTCTGTTGGTTTGTGAATTCTCTTGTATTGTTCTAATCACTGTTTCTGA
ATTTGATTCGGGTTTTTATTGAATTCTTTTTATGTGTTTTGGTATTTGCAGGT

>At.GSI21_splicesite

ACTTGGGCTTCTTCTCTCTCTATCCTCTCTCTTCTGATCTCGATTTGTTTTTTTCGAATCGCTC
TACTTCCAGTTAGATTCTTGATTTGAGATTAATTAGATTGATTATTCTAATCGTTTTTTTTGTA
AGCAATTAAGATTTATCTTGTTTTATGTTTTTCTTTAGGTATGACTTGTTATGTATGTCACTGT
TTCAGATCTGATCCTCTCTGTTGGTTTGTGAATTCTCTTGTATTGTTCTAATCACTGTTTCTGA
ATTTGATTCGGGTTTTTATTGAATTCTTTTTATGTGTTTTGGTATTGTACTTG

>Zm.GST7

ATGTCTGCTGCGGCGGCCTTCACAGTTTGTTTATTTCCTACTGTTTGCTGCGGCGATTGTTGTT
GTTTTCTGTTTTATAAATAATAAAGGAGGAGGAGATTTGTTTTGGTTTGTGTTTGTTTCCATCC
TTGCTGCTCCATCACACTATCTGTAATTTGTAAACAGCGACAATAAATAAATTAATAAATTTGG
TTTCTCATACCTATATGTGTCTGTTTGGAGGCTTGTTTGTTTGAGACATCTGTCTGGTTGTTTT
TTTGCTGCCAGCCGGTAGTATAAATTTTGTTTTTGGACGACGAA

>Zm.GST7_NUE

ATGTCTGCTGCGGCGGCCTTCACAGTTTGTTTATTTCCTACTGTTTGCTGCGGCGATTGTTGTT
GTTTTCTGTTTTATACCGCCTAAAGGAGGAGGAGATTTGTTTTGGTTTGTGTTTGTTTCCATCC
TTGCTGCTCCATCACACTATCTGTAATTTGTAAACAGCGACCCGCCATAAATTCCGCCATTTGG
TTTCTCATACCTATATGTGTCTGTTTGGAGGCTTGTTTGTTTGAGACATCTGTCTGGTTGTTTT
TTTGCTGCCAGCCGGTAGTATAAATTTTGTTTTTGGACGACGAA

>Zm.GST7_T-rich_tracts

ATGTCTGCTGCGGCGGCCGGCACAGGGTGGGTAGGTCCTACTGGGTGCTGCGGCGAGGGGGGGG
GGGGGCTGGGGGATAAATAATAAAGGAGGAGGAGAGGTGGGGGGGGGTGTGGGTGGGTCCATCC
GGGCTGCTCCATCACACTATCTGTAAGGTGTAAACAGCGACAATAAATAAAGGAATAAAGGTGG
GGTCTCATACCTATATGTGTCTGGGTGGAGGCGGGGGTGGGTGAGACATCTGTCTGGGGGGGGG
GGTGCTGCCAGCCGGTAGTATAAAGGGGGGGGGTGGACGACGAA

>Reference_expression_cassette for soybean
(At.Cyco_promoter_leader : At.Cyco_intron: Ec.uidA+St.LS1 :
Gb.Fbl2_3'UTR)

TGCGAGTGGGCGAATTCCGGAGCACTCTGATTGGCTGAAAAAATAGAAATAGTAGTGATGTTGC
TCCTCCTCTCCTCCTCTATTATTAATTTTTCGTCGTTCTTCTTCTGAAAGTTGTGTGGTTTTTA
GAGGTCACCAAAAAAAATCTATTTTGAGATACTAAAAATATTTCGTTTTGCATTTTGTTGTGCA
GCCATTTGTTACACAGGTTGAAGCTTATAACTGAAAATTGGATTCAAAGAATCGTAGATGAAGA

AATCGAAGTGAGTTGAATATTTTCTGAACATATGAAAATTGGAACAAGTTTTTTCTCATTTTGC
TAGTTTCCTGTTTTTATGTTTTCTTGACTTTAGGAGATGACATATGGAGGTGAACTATACAAAG
GTTGTTGCAACGATAACATTCTCCTTAATTCAGTTTTTGCAACTCGGTTACAAGCACTCAGTGG
ACTTTTGGCCAAGACAATTTTTTTTTTTTTTTTCTCTCTCTCTAAAATGTTATAGATACGAATC
CTTTGTTGAATAAAGGAAAAAGTTGAACATTTGATTACACATAAGACTTTAACATAATCCAACT
TTTTTTTATATGAAGCTACAAACAAGATTTAAAACATCAAAGATTCCATCTAAACTTCATTCAT
CTTCAATCTTCAACATCCTTCAATGACTAGTATGTATGTACATAAGTAAAATTGTTGATAAGAA
AACAAAACAATGATGGGCTAAAATAGCCCATAAAAGGCCCATTAAACTTGGGTTTAGACTTTAG
ATTCAACGACGCCAGATTAGTGAGTCACATAACCCTCTTGGAAAGAGTCTCAACACTTGCAGAG
AAAAAGAACAAGGAAGATCCCGGAAACAGGTAATTTCTCTCCTCTCTATTTTTACCATTTTCCA
TTGACGACGATCTAGGTTTTCTGATTTGATTTTGGAGAACGCCTCGATGAGTTTATAGATTCGT
AGATTGGTTTTGAGATTCAGTATAATTTCACCCGGATTCCAATTTTTGAACCGATACCTAATTT
TGAATTGATTTGGTAGATCGATTGGTCAAATTTGAAATTGATTTTTCTCCATAATATCTGAAGC
GTCTTATTGGATCAAATCTACAACATTTCTCTGTTGAAAGGATCGATTTTTTTTTTCTTGGAAC
ATGATAACTTTTGATTATTCATCAAAGTTTTGTTCTTTTTAATATTTCACAGGTGGTACCCTCA
GCGCTGTGCCTGTTGCGATCGCACCATGGTCCGTCCTGTAGAAACCCCAACCCGTGAAATCAAA
AAACTCGACGGCCTGTGGGCATTCAGTCTGGATCGCGAAAACTGTGGAATTGATCAGCGTTGGT
GGGAAAGCGCGTTACAAGAAAGCCGGGCAATTGCTGTGCCAGGCAGTTTTAACGATCAGTTCGC
CGATGCAGATATTCGTAATTATGCGGGCAACGTCTGGTATCAGCGCGAAGTCTTTATACCGAAA
GGTTGGGCAGGCCAGCGTATCGTGCTGCGTTTCGATGCGGTCACTCATTACGGCAAAGTGTGGG
TCAATAATCAGGAAGTGATGGAGCATCAGGGCGGCTATACGCCATTTGAAGCCGATGTCACGCC
GTATGTTATTGCCGGGAAAAGTGTACGTAAGTTTCTGCTTCTACCTTTGATATATATATAATAA
TTATCATTAATTAGTAGTAATATAATATTTCAAATATTTTTTTCAAAATAAAAGAATGTAGTAT
ATAGCAATTGCTTTTCTGTAGTTTATAAGTGTGTATATTTTAATTTATAACTTTTCTAATATAT
GACCAAAATTTGTTGATGTGCAGGTATCACCGTTTGTGTGAACAACGAACTGAACTGGCAGACT
ATCCCGCCGGGAATGGTGATTACCGACGAAAACGGCAAGAAAAAGCAGTCTTACTTCCATGATT
TCTTTAACTATGCCGGAATCCATCGCAGCGTAATGCTCTACACCACGCCGAACACCTGGGTGGA
CGATATCACCGTGGTGACGCATGTCGCGCAAGACTGTAACCACGCGTCTGTTGACTGGCAGGTG
GTGGCCAATGGTGATGTCAGCGTTGAACTGCGTGATGCGGATCAACAGGTGGTTGCAACTGGAC
AAGGCACTAGCGGGACTTTGCAAGTGGTGAATCCGCACCTCTGGCAACCGGGTGAAGGTTATCT
CTATGAACTGTGCGTCACAGCCAAAAGCCAGACAGAGTGTGATATCTACCCGCTTCGCGTCGGC
ATCCGGTCAGTGGCAGTGAAGGGCGAACAGTTCCTGATTAACCACAAACCGTTCTACTTTACTG
GCTTTGGTCGTCATGAAGATGCGGACTTGCGTGGCAAAGGATTCGATAACGTGCTGATGGTGCA
CGACCACGCATTAATGGACTGGATTGGGGCCAACTCCTACCGTACCTCGCATTACCCTTACGCT
GAAGAGATGCTCGACTGGGCAGATGAACATGGCATCGTGGTGATTGATGAAACTGCTGCTGTCG
GCTTTAACCTCTCTTTAGGCATTGGTTTCGAAGCGGGCAACAAGCCGAAAGAACTGTACAGCGA
AGAGGCAGTCAACGGGGAAACTCAGCAAGCGCACTTACAGGCGATTAAAGAGCTGATAGCGCGT
GACAAAAACCACCCAAGCGTGGTGATGTGGAGTATTGCCAACGAACCGGATACCCGTCCGCAAG
GTGCACGGGAATATTTCGCGCCACTGGCGGAAGCAACGCGTAAACTCGACCCGACGCGTCCGAT
CACCTGCGTCAATGTAATGTTCTGCGACGCTCACACCGATACCATCAGCGATCTCTTTGATGTG
CTGTGCCTGAACCGTTATTACGGATGGTATGTCCAAAGCGGCGATTTGGAAACGGCAGAGAAGG
TACTGGAAAAAGAACTTCTGGCCTGGCAGGAGAAACTGCATCAGCCGATTATCATCACCGAATA
CGGCGTGGATACGTTAGCCGGGCTGCACTCAATGTACACCGACATGTGGAGTGAAGAGTATCAG
TGTGCATGGCTGGATATGTATCACCGCGTCTTTGATCGCGTCAGCGCCGTCGTCGGTGAACAGG
TATGGAATTTCGCCGATTTTGCGACCTCGCAAGGCATATTGCGCGTTGGCGGTAACAAGAAAGG
GATCTTCACTCGCGACCGCAAACCGAAGTCGGCGGCTTTTCTGCTGCAAAAACGCTGGACTGGC
ATGAACTTCGGTGAAAAACCGCAGCAGGGAGGCAAACAATGAGCGATCGCCAGCAGAACACGCG

CTGAGGTTAATTAAACCATATGACACTGGTGCATGTGCCATCATCATGCAGTAATTTCATGGTA
TATCTTAATTATATGGTTAATAAAAAAAAGATGGTGAGTGAATAATGTGCGTGCATTCCTCCAT
GCACCAATGGTGAATCTCTTTGCATACATAGAGATTCTGAATGATTATAGTTTATGTTGTAGTG
AAATTAATTTTGAATGTTGTTTTTAAATTTTAATGTCACTTGGCTTGATTTATGTTTTAACGAA
GCTTATGTTATGTATTTTACTTTAATGATATTGCATGTATTGTTAATTTAACATTGCTTGATCA
GTATACTCT

>At.Cyco_promoter_leader

TGCGAGTGGGCGAATTCCGGAGCACTCTGATTGGCTGAAAAAATAGAAATAGTAGTGATGTTGC
TCCTCCTCTCCTCCTCTATTATTAATTTTTCGTCGTTCTTCTTCTGAAAGTTGTGTGGTTTTTA
GAGGTCACCAAAAAAAATCTATTTTGAGATACTAAAAATATTTCGTTTTGCATTTTGTTGTGCA
GCCATTTGTTACACAGGTTGAAGCTTATAACTGAAAATTGGATTCAAAGAATCGTAGATGAAGA
AATCGAAGTGAGTTGAATATTTTCTGAACATATGAAAATTGGAACAAGTTTTTTCTCATTTTGC
TAGTTTCCTGTTTTTATGTTTTCTTGACTTTAGGAGATGACATATGGAGGTGAACTATACAAAG
GTTGTTGCAACGATAACATTCTCCTTAATTCAGTTTTTGCAACTCGGTTACAAGCACTCAGTGG
ACTTTTGGCCAAGACAATTTTTTTTTTTTTTTTCTCTCTCTCTAAAATGTTATAGATACGAATC
CTTTGTTGAATAAAGGAAAAAGTTGAACATTTGATTACACATAAGACTTTAACATAATCCAACT
TTTTTTTATATGAAGCTACAAACAAGATTTAAAACATCAAAGATTCCATCTAAACTTCATTCAT
CTTCAATCTTCAACATCCTTCAATGACTAGTATGTATGTACATAAGTAAAATTGTTGATAAGAA
AACAAAACAATGATGGGCTAAAATAGCCCATAAAAGGCCCATTAAACTTGGGTTTAGACTTTAG
ATTCAACGACGCCAGATTAGTGAGTCACATAACCCTCTTGGAAAGAGTCTCAACACTTGCAGAG
AAAAAGAACAAGGAAGATCCCGGAAA

>At.Cyco_intron

CAGGTAATTTCTCTCCTCTCTATTTTTACCATTTTCCATTGACGACGATCTAGGTTTTCTGATT
TGATTTTGGAGAACGCCTCGATGAGTTTATAGATTCGTAGATTGGTTTTGAGATTCAGTATAAT
TTCACCCGGATTCCAATTTTTGAACCGATACCTAATTTTGAATTGATTTGGTAGATCGATTGGT
CAAATTTGAAATTGATTTTTCTCCATAATATCTGAAGCGTCTTATTGGATCAAATCTACAACAT
TTCTCTGTTGAAAGGATCGATTTTTTTTTTCTTGGAACATGATAACTTTTGATTATTCATCAAA
GTTTTGTTCTTTTTAATATTTCACAGGT

>Gb.Fbl2_3’UTR

ACCATATGACACTGGTGCATGTGCCATCATCATGCAGTAATTTCATGGTATATCTTAATTATAT
GGTTAATAAAAAAAAGATGGTGAGTGAATAATGTGCGTGCATTCCTCCATGCACCAATGGTGAA
TCTCTTTGCATACATAGAGATTCTGAATGATTATAGTTTATGTTGTAGTGAAATTAATTTTGAA
TGTTGTTTTTAAATTTTAATGTCACTTGGCTTGATTTATGTTTTAACGAAGCTTATGTTATGTA
TTTTACTTTAATGATATTGCATGTATTGTTAATTTAACATTGCTTGATCAGTATACTCT

>Zm.GST43

TCCAGGGCGCCTGCTTGTTTGCTGCCAAGAGAGTGTTTTGTGTACTGCTGCTGCCGAGAAATAT
ATATTTTTTTCTTTCACCACCTCGTGTGCAGCAGTTGTTTTTTGTTTGGATGGATAAATGTTTC
TAGTACTGTGGAGGCTGCATCTGCATCTGTTTGTAAATGGATGAAATATGAATAAAAAGTTTTG

TTTCTCATACCCCATGTGTCTTGTGTTTGCATGCACGCCGTGCTAGTTTGGTTTTTGGTTTCTA
GAGAAAACATTTTGTTTGCTTGTTTCTCTATAGGATGTGAAGAA

>Reference_expression_cassette for maize (35S_promoter : Zm.DNAK_intron : Ec.uidA+St.LS1 : Sb.Nltp_3'UTR)

AGATTAGCCTTTTCAATTTCAGAAAGAATGCTAACCCACAGATGGTTAGAGAGGCTTACGCAGC
AGGTCTCATCAAGACGATCTACCCGAGCAATAATCTCCAGGAAATCAAATACCTTCCCAAGAAG
GTTAAAGATGCAGTCAAAAGATTCAGGACTAACTGCATCAAGAACACAGAGAAAGATATATTTC
TCAAGATCAGAAGTACTATTCCAGTATGGACGATTCAAGGCTTGCTTCACAAACCAAGGCAAGT
AATAGAGATTGGAGTCTCTAAAAAGGTAGTTCCCACTGAATCAAAGGCCATGGAGTCAAAGATT
CAAATAGAGGACCTAACAGAACTCGCCGTAAAGACTGGCGAACAGTTCATACAGAGTCTCTTAC
GACTCAATGACAAGAAGAAAATCTTCGTCAACATGGTGGAGCACGACACACTTGTCTACTCCAA
AAATATCAAAGATACAGTCTCAGAAGACCAAAGGGCAATTGAGACTTTTCAACAAAGGGTAATA
TCCGGAAACCTCCTCGGATTCCATTGCCCAGCTATCTGTCACTTTATTGTGAAGATAGTGGAAA
AGGAAGGTGGCTCCTACAAATGCCATCATTGCGATAAAGGAAAGGCCATCGTTGAAGATGCCTC
TGCCGACAGTGGTCCCAAAGATGGACCCCCACCCACGAGGAGCATCGTGGAAAAAGAAGACGTT
CCAACCACGTCTTCAAAGCAAGTGGATTGATGTGATATCTCCACTGACGTAAGGGATGACGCAC
AATCCCACTATCCTTCGCAAGACCCTTCCTCTATATAAGGAAGTTCATTTCATTTGGAGAGGAC
ACGGGGGACTCTAGAGGATCCACTAGTCGGACCGACCGTCTTCGGTACGCGCTCACTCCGCCCT
CTGCCTTTGTTACTGCCACGTTTCTCTGAATGCTCTCTTGTGTGGTGATTGCTGAGAGTGGTTT
AGCTGGATCTAGAATTACACTCTGAAATCGTGTTCTGCCTGTGCTGATTACTTGCCGTCCTTTG
TAGCAGCAAAATATAGGGACATGGTAGTACGAAACGAAGATAGAACCTACACAGCAATACGAGA
AATGTGTAATTTGGTGCTTAGCGGTATTTATTTAAGCACATGTTGGTGTTATAGGGCACTTGGA
TTCAGAAGTTTGCTGTTAATTTAGGCACAGGCTTCATACTACATGGGTCAATAGTATAGGGATT
CATATTATAGGCGATACTATAATAATTTGTTCGTCTGCAGAGCTTATTATTTGCCAAAATTAGA
TATTCCTATTCTGTTTTTGTTTGTGTGCTGTTAAATTGTTAACGCCTGAAGGAATAAATATAAA
TGACGAAATTTTGATGTTTATCTCTGCTCCTTTATTGTGACCATAAGTCAAGATCAGATGCACT
TGTTTTAAATATTGTTGTCTGAAGAAATAAGTACTGACAGTATTTTGATGCATTGATCTGCTTG
TTTGTTGTAACAAAATTTAAAAATAAAGAGTTTCCTTTTTGTTGCTCTCCTTACCTCCTGATGG
TATCTAGTATCTACCAACTGACACTATATTGCTTCTCTTTACATACGTATCTTGCTCGATGCCT
TCTCCCTAGTGTTGACCAGTGTTACTCACATAGTCTTTGCTCATTTCATTGTAATGCAGATACC
AAGCGGGGTACCCTCAGCGCTGTGCCTGTTGCGATCGCACCATGGTGAGGCCCGTTGAGACCCC
GACTAGGGAGATCAAGAAGCTGGACGGCCTCTGGGCCTTCTCCCTCGACCGTGAGAACTGCGGC
ATCGACCAGCGCTGGTGGGAGTCCGCCCTCCAGGAGTCTAGGGCCATCGCCGTGCCCGGTTCCT
TCAACGACCAGTTCGCCGACGCCGACATCCGCAACTACGCGGGCAACGTCTGGTATCAGCGCGA
GGTGTTCATCCCGAAGGGCTGGGCGGGCCAGCGCATCGTGCTCCGCTTCGACGCCGTGACCCAC
TACGGCAAGGTCTGGGTGAACAATCAGGAGGTAAGTTTCTGCTTCTACCTTTGATATATATATA
ATAATTATCATTAATTAGTAGTAATATAATATTTCAAATATTTTTTTCAAAATAAAAGAATGTA
GTATATAGCAATTGCTTTTCTGTAGTTTATAAGTGTGTATATTTTAATTTATAACTTTTCTAAT
ATATGACCAAAATTTGTTGATGTGCAGGTGATGGAGCACCAGGGCGGTTACACCCCGTTCGAGG
CCGACGTGACGCCGTACGTGATCGCCGGGAAGTCCGTCCGCATCACCGTCTGCGTGAACAATGA
GCTGAACTGGCAGACCATCCCGCCTGGCATGGTCATCACCGACGAGAACGGCAAGAAGAAGCAG
TCCTACTTCCACGACTTCTTCAACTACGCTGGCATCCACCGCTCCGTGATGCTCTACACCACTC
CCAACACCTGGGTGGACGACATCACCGTGGTCACCCACGTGGCCCAGGACTGCAACCACGCCTC
CGTGGACTGGCAAGTCGTTGCCAACGGCGACGTCAGCGTCGAGCTGCGCGACGCCGACCAGCAA
GTCGTTGCCACCGGCCAGGGCACCAGCGGCACCCTCCAAGTCGTCAACCCTCACCTCTGGCAGC

CTGGCGAGGGCTACCTCTACGAGCTGTGCGTCACCGCCAAGAGCCAGACTGAGTGCGACATCTA
CCCTCTCCGCGTCGGCATCAGGAGCGTCGCTGTCAAGGGCGAGCAGTTCCTCATCAACCACAAG
CCTTTCTACTTCACTGGTTTCGGCCGCCACGAGGACGCTGACCTGAGGGGCAAGGGTTTCGACA
ACGTCCTGATGGTCCACGACCACGCTCTGATGGACTGGATCGGTGCCAACAGCTACAGGACCAG
TCACTACCCGTACGCTGAGGAGATGCTGGACTGGGCTGACGAGCACGGTATCGTCGTGATCGAC
GAGACTGCTGCGGTCGGTTTCAACCTGTCTCTGGGCATTGGTTTCGAGGCTGGGAACAAGCCGA
AGGAGCTGTACTCTGAGGAAGCTGTCAACGGCGAGACTCAGCAAGCTCATCTCCAGGCGATTAA
GGAGCTGATTGCCAGGGACAAGAACCATCCGTCTGTCGTGATGTGGTCTATTGCGAATGAGCCG
GACACCAGACCGCAAGGGGCGCGTGAATACTTCGCGCCGCTGGCGGAGGCGACTCGCAAACTGG
ACCCAACCCGTCCAATCACGTGCGTCAATGTCATGTTCTGCGACGCCCATACGGATACGATCTC
GGACCTGTTCGATGTTCTTTGTCTCAATCGGTACTATGGGTGGTATGTTCAGAGCGGGGATCTT
GAGACGGCGGAGAAGGTTCTTGAGAAGGAACTCCTGGCGTGGCAAGAGAAGCTCCATCAGCCGA
TCATTATCACGGAGTACGGGGTTGACACACTTGCGGGCCTTCACAGTATGTACACAGATATGTG
GTCGGAGGAATACCAGTGTGCATGGTTGGATATGTACCATCGTGTCTTCGACCGGGTTTCAGCG
GTTGTCGGCGAACAAGTCTGGAACTTCGCAGACTTCGCCACGAGCCAAGGGATACTGCGGGTAG
GAGGGAACAAGAAGGGAATCTTCACACGGGATCGGAAGCCCAAGTCAGCAGCCTTCCTGTTGCA
GAAGCGATGGACAGGAATGAACTTCGGAGAAAAGCCACAGCAAGGCGGAAAGCAGTGAGCGATC
GCCAGCAGAACACGCGCTGAGGTTAATTAAGGGCCCTGATCGATCATAAGGACATGCATGAGGC
ATGCTCGTTGGATGGCGATCAGCATGCAGTCGTCGTTGTTTTACCAGATGTCGTCCATGACTCC
CTCCATGGATGATGCATGCAATGAGATAGATACATAAATTAAGGAGTAGATCATGCATGCACCG
TACCTGTTAGTGATCACTCCGTTTAATTAGTATTTACTGTTTACTACCTTGAGTTTACACCAAC
TCTGTCGTCGCTGTGGCTGTCGTCGTGTACCTCGATCGTACGTGTGTGTGTGTAGCAGGCTAGC
AAAGCAGCACTCAACGTCGTCCTTGGGGGTTTGCTTGCCTTTAACCTTGTTGCTTTAGCACACA
GGTTTCTGAATAATGAATACTACTAGCTTTTGTGTCCATTATATATATATTGTACTGTATGCAT
GATGTTTTCCATCCATGAGTCATGAGAGTGATGTGTTCAAGTAATAAGATATAGTAACATATAC
ACGCGACGTAC

> Sb.Nltp4_3'UTR

TGATCGATCATAAGGACATGCATGAGGCATGCTCGTTGGATGGCGATCAGCATGCAGTCGTCGT
TGTTTTACCAGATGTCGTCCATGACTCCCTCCATGGATGATGCATGCAATGAGATAGATACATA
AATTAAGGAGTAGATCATGCATGCACCGTACCTGTTAGTGATCACTCCGTTTAATTAGTATTTA
CTGTTTACTACCTTGAGTTTACACCAACTCTGTCGTCGCTGTGGCTGTCGTCGTGTACCTCGAT
CGTACGTGTGTGTGTGTAGCAGGCTAGCAAAGCAGCACTCAACGTCGTCCTTGGGGGTTTGCTT
GCCTTTAACCTTGTTGCTTTAGCACACAGGTTTCTGAATAATGAATACTACTAGCTTTTGTGTC
CATTATATATATATTGTACTGTATGCATGATGTTTTCCATCCATGAGTCATGAGAGTGATGTGT
TCAAGTAATAAGATATAGTAACATATACACGCGACGTAC

S2 Primer/ probe lists

S2.1 5’ RACE gene specific primers (nested):

Outer: CGAATATCTGCATCGGCGAACTGATC

Inner: TGGCACAGCAATTGCCCGGC

S2.2 Gene specific primer for generating amplicons for high throut sequencing:

For Intron splicing and TSS characterization: TGCCAGTTCAGTTCGTTGTTC

For 3’ polyadenylation site characterization: CTCGCAAGGCATATTGCGCGTT

S2.3 qRT-PCR TaqMan assay primer-probes for GUS and readthrough analysis:

Testing in Soybeans:

Normalizing gene:

Glyma13g27570

Forward Primer	Reverse Primer	Probe
GGGCCGCAGTCCTTCAA	CCACCATTCCACTGGTTTGG	CAAACAGGCTCAGGCAG

Glyma17g17790

Forward Primer	Reverse Primer	Probe
CAGCGCTACAGAACCCAGATC	GACGCGTGCCTTGGACAT	CGGGTTCCAATCGTCGA

GUS transcript:

Forward Primer	Reverse Primer	Probe
ACCGAATACGGCGTGGATAC	TCCAGCCATGCACACTGATAC	TGTACACCGACATGTGG

Readthrough:

Forward Primer	Reverse Primer	Probe
ACAGGGTAATAGGTCTCACGCG	GCCGCAAATCAACCTCACTC	CAAATCCTACCACCTCATTTA

Testing in Maize:

Normalizing gene 18S:

Forward Primer	Reverse Primer	Probe
CGTCCCTGCCCTTTGTACAC	CGAACACTTCACCGGATCATT	CCGCCCGTCGCTCCTACCGAT

GUS transcript:

Forward Primer	Reverse Primer	Probe
CGACGCCCATACGGATACGAT	CCGCTCTGAACATACCACCCATAG TA	TCGGACCTGTTCGATGTTCTTTGTC TCAATCG

Readthrough:

Forward Primer	Reverse Primer	Probe
CTAGGGATAACAGGGTAATAGGT CTCA	CGCAAATCAACCTCACTCTATTTA AA	CGGCAAATCCTACCACC

S3 Supplementary Table 1. Expression element BLAST search against Arabidopsis and maize genome sequences

BLAST results against Arabidopsis TAIR9	Total Sequence Length	Number of BLAST alignments with E-value <1	Highest scoring alignment					
			Score	Expect	Alignment Length	Identities	Gaps	Longest continuous match (bp)
At.Cyco_promoter_leader	858	82	1540.5	0E+00	858	99%	0%	465
At.Cyco_intron	348	33	620.0	1E-176	343	100%	0%	343
Gb.Fbl2_3'UTR	315	36	41.0	2E-02	22	100%	0%	22
At.GSP442	500	12	44.6	2E-03	44	81%	11%	16
At.GSP571	500	32	50.0	5E-05	98	73%	3%	13
At.GSP576	500	22	44.6	2E-03	40	85%	8%	14
At.GSI17	300	24	41.9	2E-02	30	90%	0%	13
At.GSI21	309	30	46.4	4E-04	35	88%	0%	15
Zm..GST7	300	10	41.0	2E-02	41	82%	5%	17
Zm.GST43	300	12	37.4	2E-01	20	100%	0%	20
**BLAST results against maize B73v1**	**Total Sequence Length**	**Number of BLAST alignments with E-value <1**	**Highest scoring alignment**					
			Score	Expect	Alignment Length	Identities	Gaps	Longest continuous match (bp)
At.Cyco_promoter_leader	858	10	42.8	2E-01	29	90%	6%	14
At.Cyco_intron	348	19	41.0	3E-01	29	93%	3%	14
Gb.Fbl2_3'UTR	315	12	44.6	2E-02	34	88%	0%	14
At.GSP442	500	12	41.9	4E-01	30	90%	0%	12
At.GSP571	500	8	51.8	2E-04	72	77%	10%	16
At.GSP576	500	7	42.8	1E-01	28	92%	0%	14
At.GSI17	300	10	42.8	7E-02	63	77%	8%	11
At.GSI21	309	10	41.0	3E-01	43	81%	7%	16
Zm..GST7	300	8	41.9	3E-01	35	85%	0%	20
Zm.GST43	300	16	41.0	3E-01	32	87%	0%	28

S3.1 Methods

Expression elements were analyzed for matches against the Arabidopsis TAIR9 (Swarbreck et al., 2008) and Maize B73 RefGen_v1 (Schnable et al., 2009) genome assemblies by using Basic Local Alignment Search Tool BLASTN 2.9 (Altschul et al., 1997) with default parameters: word size (11) and score (match 2/mismatch-3) with gap costs (existence 5/extension 2).

S3.2 Results: New expression elements do not have significant sequence matches to native plant sequences

A single gap was detected within in a poly T tract in At.Cyco_promoter_leader, which can likely be attributed to a minor difference in the sequencing of either the construct or reference genome.

A two base and three base mismatch was identified at the 5' and 3' end of the At.Cyco_intron element, which can be explained by the nucleotide modifications that were introduced in the 5'and 3' exonic ends flanking the intron to facilitate cloning. Other than these minor differences, the highest scoring alignments for both elements from Arabidopsis were otherwise identical to the sequence in TAIR9 reference genome.

BLAST searches with the native cotton sequence Gb.Fbl2_3'UTR generated 36 matches to the Arabidopsis genome with *E*-score<1, but closer examination of these matches revealed that these only represented isolated short stretches of sequence identity. For example, the highest scoring alignment was a 22bp match with low score= 41 and low significance (*E*-score e-2). Similarly, BLAST searches with the native Arabidopsis sequences At.Cyco_promoter+leader and At.Cyco_intron, as well as the native cotton sequence Gb.Fbl2_3'UTR, against the maize genome only produced a small number (<19) of BLAST alignments with *E*-score<1 and all of these consisted of short alignments (<34 bp) with low scores (<45) and significance (*E*-score ≥ e-2).

The new expression elements that are computationally derived from Arabidopsis or maize sequences only generated matches to the Arabidopsis or maize genomic sequences with low scores (score <52). Generally the sequence alignments were short, and comprised a small portion of the new sequence. Among all computationally derived elements, At.GSP571 was found to have the longest alignment (98 bp) to the promoter of an Arabidopsis gene, which can be a results of the computational methods used on the training set. In addition, the alignment was gapped with <13 bp contiguous nucleotide matches and 73% overall sequence identity, indicating that the At.GSP571 sequence is all together novel and distinct from any Arabidopsis sequence. No matches of significance to the Arabidopsis or maize genomes were found with other computationally derived elements (lowest *E*-score e-4).

Overall these results indicate that new expression element sequences bear no significant sequence identity to Arabidopsis or maize genomic sequences and provide opportunities to diversify expression element sequences.

S4 Supplementary Table 2. Promoter TSS mapping by high throughput sequencing

Promoter	Intron	Total trimmed reads mapped to cassette	TSS position	No. of trimmed reads mapped to TSS position	% TSS reads mapped to TSS position
At.GSP571	At.Cyco_intron	33425	452	1747	28.5
At.GSP571	At.Cyco_intron		455	2539	41.4
At.GSP571	At.Cyco_intron		459	1846	30.1
At.GSP571	At.GSI21	129075	452	7930	33.1
At.GSP571	At.GSI21		455	10203	42.6
At.GSP571	At.GSI21		459	5833	24.3
At.GSP571	At.GSI21_IME	105944	452	6346	32.3
At.GSP571	At.GSI21_IME		455	8504	43.3
At.GSP571	At.GSI21_IME		459	4794	24.4
At.GSP571	At.GSI21_splicesite	45606	452	2806	40.0
At.GSP571	At.GSI21_splicesite		455	2421	34.5
At.GSP571	At.GSI21_splicesite		459	1780	25.4
At.GSP576	At.Cyco_intron	138720	459	10679	51.5
At.GSP576	At.Cyco_intron		462	10033	48.4
At.GSP576	At.GSI17	326046	459	32363	53.1
At.GSP576	At.GSI17		462	28531	46.9
At.GSP576	At.GSI17_IME	226385	459	23560	55.3
At.GSP576	At.GSI17_IME		462	18429	43.3
At.GSP576	At.GSI17_splicesite	187265	459	11333	46.3
At.GSP576	At.GSI17_splicesite		462	13099	53.5

S4.1 Methods

Plant materials from soy plants stably transformed with expression cassettes 3, 7, 10, 11, 4, 8, 12, and 13 were generated as described in the methods. Sequencing reads were generated and mapped to the cassettes as described in the intron splicing and 3' polyadenylation characterization methods. 5' TSS reads were identified based on alignment of reads containing the 5' sequencing adapter. A total of twelve events per cassette were analyzed.

S4.2 Results

The TSS for At.GSP571 and At.GSP576 were found to be concentrated within a narrow window of <10 bp. Three specific TSS and two specific TSS were identified for At.GSP571 and At.GSP576, respectively. These TSS's overlap with the results from the 5' RACE analysis and occur in Inr-like motifs, with CA at the -1 position. The mapped TSS for each promoter are consistent across different introns, indicating that the transcription initiation signals are robust and predictable.

S5 Supplementary Figure 1. Zm.GST analysis in maize

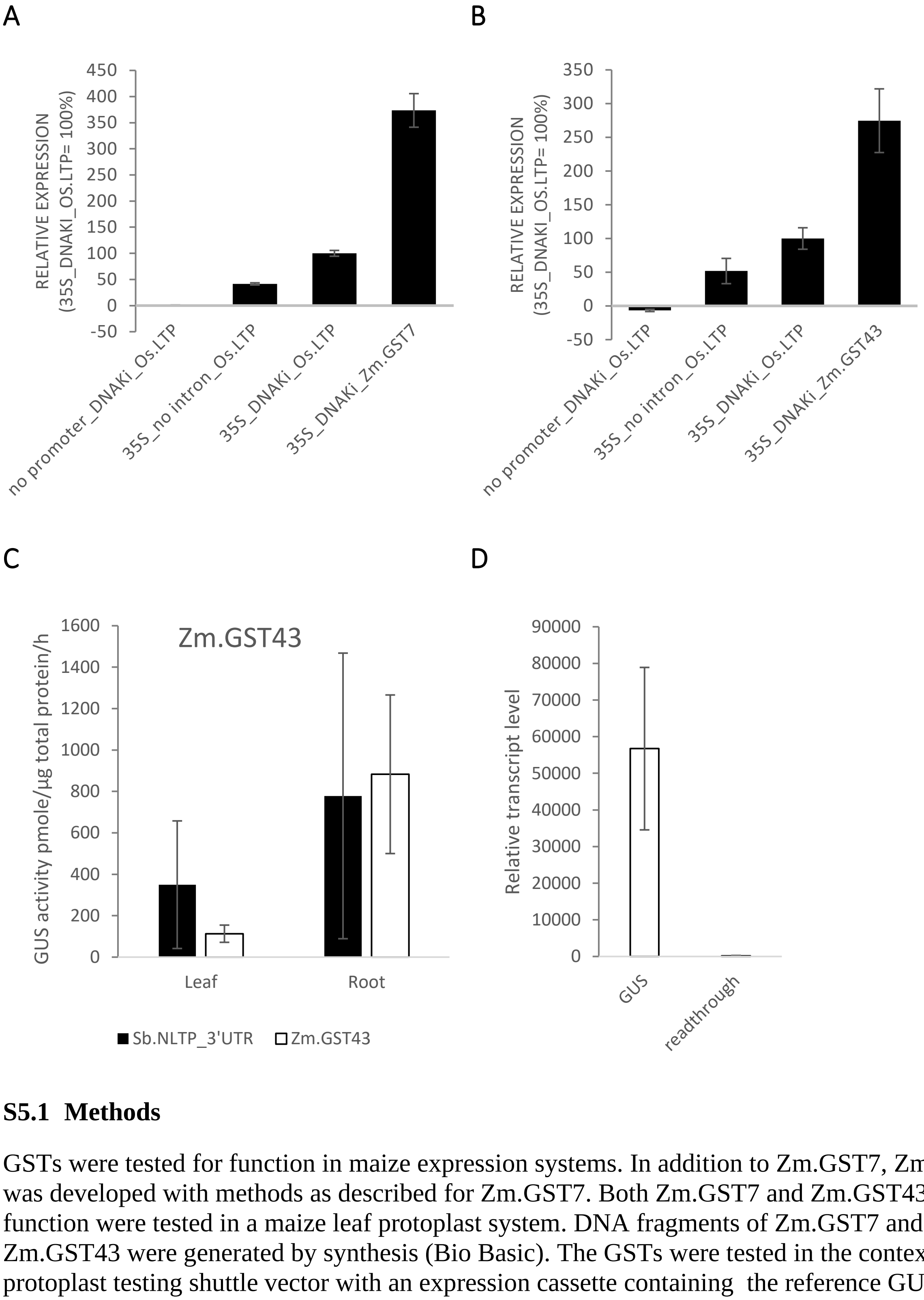

S5.1 Methods

GSTs were tested for function in maize expression systems. In addition to Zm.GST7, Zm.GST43 was developed with methods as described for Zm.GST7. Both Zm.GST7 and Zm.GST43 function were tested in a maize leaf protoplast system. DNA fragments of Zm.GST7 and Zm.GST43 were generated by synthesis (Bio Basic). The GSTs were tested in the context of a protoplast testing shuttle vector with an expression cassette containing the reference GUS expression cassette, constructed as follows from 5’ to 3’: the promoter and leader from

Cauliflower mosaic virus 35S gene (CaMV.35S), intron from *Zea mays* heat shock protein 70 gene (Zm.DNAKi) inserted into 5’UTR, coding sequence from *Escherichia coli* GUS gene with an inserted intron from *Solanum tuberosum* light inducible gene (Ec.uidA+St.LS1), and 3’UTR from *Oryza sativa* lipid transfer protein-like (Os.LTP). Protoplast testing was performed as described in (Sheen, 1991) with in plate controls including the promoterless GUS cassette (no promoter_DNAKi_Os.LTP), the reference GUS cassette (35S_DNAKi_Os.LTP), the intronless control GUS cassette (35S_no intron_Os.LTP) with expression expected at 35-50% relative to the reference, and the test cassette with the 3’UTR of interest substituted for Os.LTP_3’UTR. Three independent experiments were performed with similar results. The results of one experiment with eight technical replicates of the protoplast transformation are shown.

In addition, Zm.GST43 was tested in the context of a reference GUS reporter gene cassette for expression testing in maize stable transformants, with a promoter from Cauliflower mosaic virus (CaMV.35S_promoter), an intron from Zea mays HSP70 DNAK gene (Zm.DNAK_intron), and a 3’UTR from *Sorghum bicolor* nonspecific lipid-transfer protein 4 (Sb.Nltp_3’UTR). To generate expression cassette to test Zm.GST43, Sb.Nltp_3’UTR was replaced by Zm.GST43 in the reference cassette. The reference cassette and the cassette containing Zm.GST43 were each inserted into a binary plant transformation vector and verified by sequencing. The T-DNA vectors were transformed into Agrobacterium and introduced into *Zea mays* by Agrobacterium-mediated transformation. Transformed plants that had a single copy of the GUS transgene were selected for further tissue sampling and analysis. Sequences of the reference expression cassette and component elements are provided in supplementary materials S1. Leaf and root tissues were sampled from transformed plants at V2 stage and assayed for GUS enzymatic activity as described in the methods. GUS transcripts and read through from GUS reporter gene with Zm.GST43 was analyzed by qRT-PCR. GUS and readthrough transcripts were detected by primer/probe sets in the GUS coding sequence and downstream of the 3’UTR, respectively, as described in the methods. Transcript levels within each tissue are normalized to a housekeeping gene by 2 (-DeltaDeltaC(T)) method and reported as a relative expression value. At least twelve independent transgenic events were analyzed and the data are reported as the mean with standard error. % readthrough is calculated by normalizing readthrough transcript to GUS transcript. Primer/Probe sequences are provided in supplementary materials S4.

S5.2 Results: New 3’UTRs drive effective expression and give proper transcript termination as predicted in maize.

In transiently transformed protoplasts, GST7 and GST43 demonstrated expression 3.7-fold and 2.7-fold, respectively, as compared to the 35S_DNAKi_Os.LTP reference construct, indicating that both 3’UTRs are active and can enable expression in maize.

In order to further study the function of Zm. GST43, the GST was tested in stably transformed maize plants. The cassette with Zm.GST43 showed comparable expression to the reference cassette with Sb.Nltp_3’UTR in both leaf and root, with a higher measured GUS reporter activity in roots than in leaf (Supplementary Figure 1C).

The levels of readthrough were near the limit of quantitation, resulting in a very low calculated % read through of 0.27%, which represent essentially no readthrough to impact neighboring genes (Supplementary Figure 1D), further validating the function of Zm.GST43 in maize.

S6 Supplementary Figure 2. PolyA mapping for GST7 variants

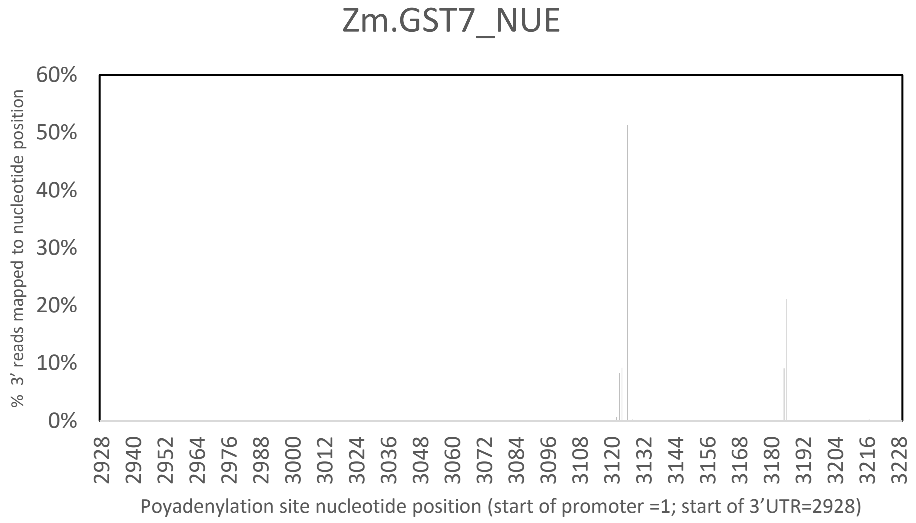

S6.1 Methods

3'Polyadenylation sites for Zm.GST7_NUE and Zm.GST7_T-rich_tracts were characterized as described in the methods section. A total of twelve events per cassette were analyzed.

S6.2 Results

A total of 574525 reads mapped to the expression cassette with Zm.GST7_NUE, of which 1610 reads aligned with the 3'sequencing adaptor sequence to identify polyadenylation sites. The polyadenylation profile for Zm.GST7_NUE was consistent with the original Zm.GST7, with dominant polyadenylation sites at 3126 and 3186 (Supplementary Figure 2). These results indicate that the mutated NUEs but did not impact polyadenylation site choice. However, the lower number of 3'sequencing adaptor reads relative to the total reads mapped to the expression cassette, compared to Zm.GST7, may indicate that the NUE mutation reduced the efficiency of the cleavage and polyadenylation, which is consistent with the overall reduction in GUS transcript levels from this expression cassette together with an increase in readthrough transcripts (Figure 8 B and C).

A total of 10654 reads mapped to the expression cassette with Zm.GST7_T_richtract, of which only 7 reads aligned with the 3'sequencing adaptor. All 7 reads mapped to the 3' end of the GUS coding sequence and none of the reads mapped within the 3'UTR, indicating that the mutation of the T-rich tracts abolished proper cleavage and transcript processing. The polyA results are also consistent with the significant reduction in transcript levels and increase in readthrough transcripts (Figure 8 B and C).

S7 Supplementary Figure 3. Comparison of new expression element cassettes with CaMV.35S promoter

A

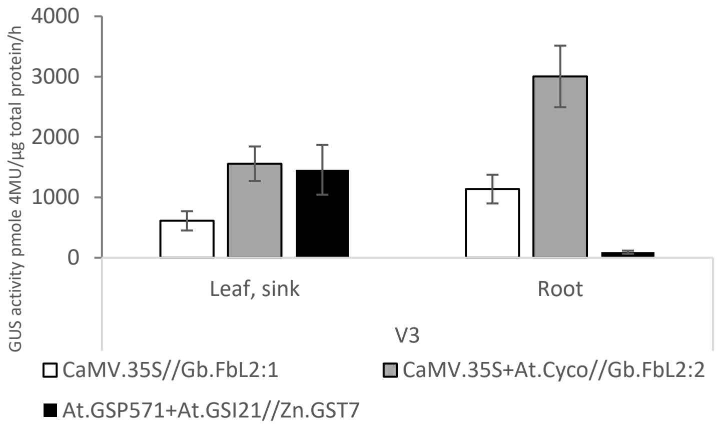

B

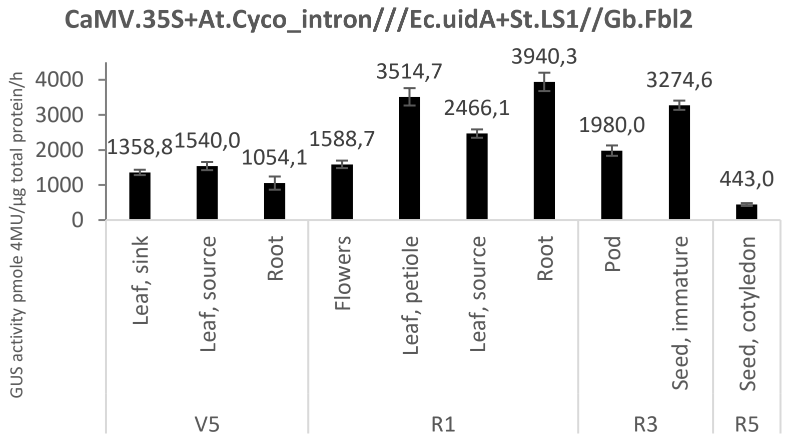

C

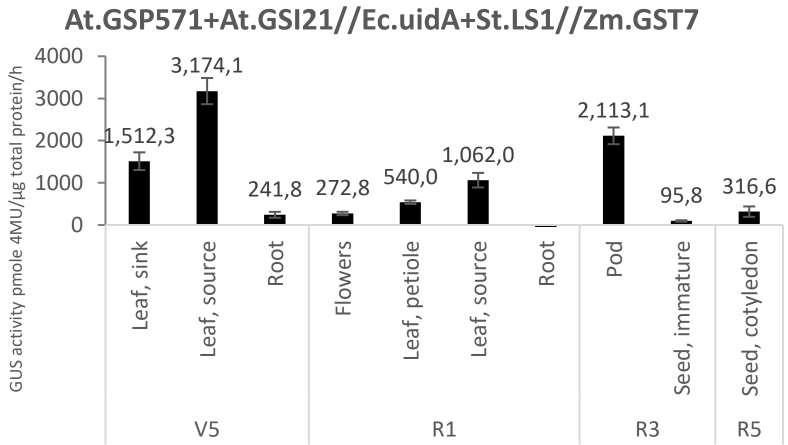

S7.1 Methods

The 3 expression cassettes were constructed as described in the methods and stably transformed into soy plants. GUS activity was measured across multiple tissue types as described in the methods. For each tissue type, >6 plants were assayed. The averages are reported in the figure with error bars representing standard error.

Cassette	Promoter	Intron	GOI	3' UTR
17	CaMV.35S_promoter_leader	-	Ec.uidA+St.LS1	Gb.Fbl2
18	CaMV.35S_promoter_leader	At.Cyco_intron	Ec.uidA+St.LS1	Gb.Fbl2
19	At.GSP571	At.GSI21	Ec.uidA+St.LS1	Zm.GST7

S7.2 Results

To evaluate if the GSP, GSI, and GST are compatible within a single cassette, cassette 19 was generated and compared to the CaMV.35S promoter that is commonly used in transgenes in cassettes 17 and 18. The results demonstrates IME from the At.Cyco intron on CaMV35S promoter. In addition, the all new expression cassette with a combination of GSP, GSI, and GST, demonstrate compatibility of these different computationally derived element types. The expression of the full cassette with GSP, GSI, and GST, delivers expression levels that are higher than CaMV35S without the enhancement of an intron in V3 leaves, but lower in root, which is aligned with the intended expression profile of At.GSP571 in leaves. In addition, GSP/GSI/ GST expression cassette can deliver expression levels that are comparable to CaMV.35S enhanced by the At.Cyco intron in leaves in vegetative stage. These results demonstrate the opportunity for these computationally derived expression elements to build new and unique expression cassettes to enable further fine tuning of expression profiles.

References cited

Altschul, S.F., Madden, T.L., Schäffer, A.A., Zhang, J., Zhang, Z., Miller, W., *et al.* (1997). Gapped BLAST and PSI-BLAST: a new generation of protein database search programs. Nucleic Acids Research 25, 3389-3402. doi: 10.1093/nar/25.17.3389

Schnable, P.S., Ware, D., Fulton, R.S., Stein, J.C., Wei, F., Pasternak, S., *et al.* (2009). The B73 Maize Genome: Complexity, Diversity, and Dynamics. Science 326, 1112-1115. doi: 10.1126/science.1178534

Sheen, J. (1991). Molecular mechanisms underlying the differential expression of maize pyruvate, orthophosphate dikinase genes. Plant Cell 3, 225-245. doi: 10.1105/tpc.3.3.225

Swarbreck, D., Wilks, C., Lamesch, P., Berardini, T.Z., Garcia-Hernandez, M., Foerster, H., *et al.* (2008). The Arabidopsis Information Resource (TAIR): gene structure and function annotation. Nucleic Acids Res 36, D1009-1014. doi: 10.1093/nar/gkm965
